# Supplementary figures and images for: Primates in peril: the significance of Brazil, Madagascar, Indonesia and the Democratic Republic of the Congo for global primate conservation
Source: PeerJ. 2018 Jun 15;6:e4869. doi: 10.7717/peerj.4869 (PMC6005167; doi:10.7717/peerj.4869)

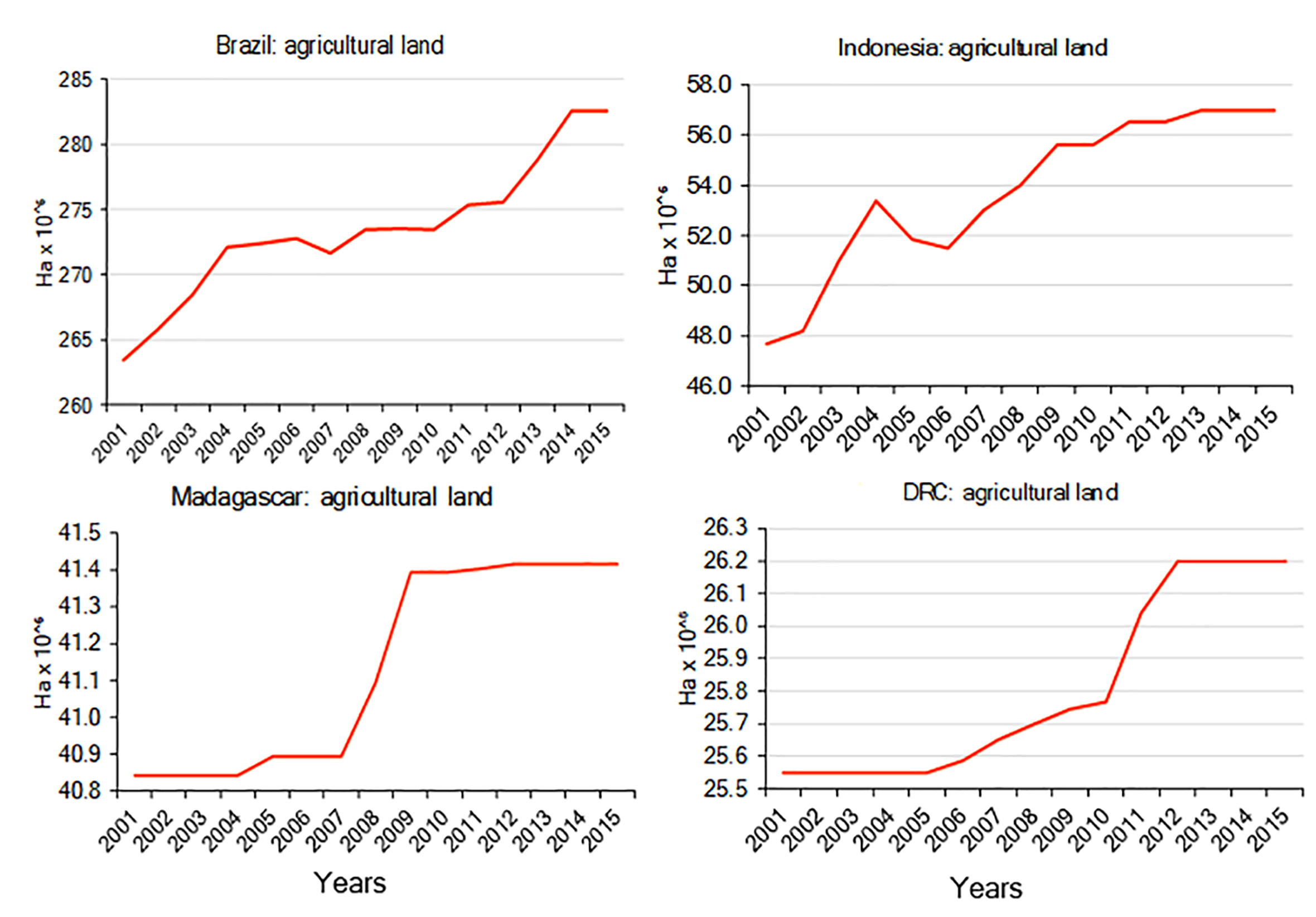

Supplement: Supplemental Information 1 — Available at FAOStats http://www.fao.org/faostat/en/#compare (accessed 10 February 2018). See Text S1 for limitations of the FAO data. [file peerj-06-4869-s001.jpg]

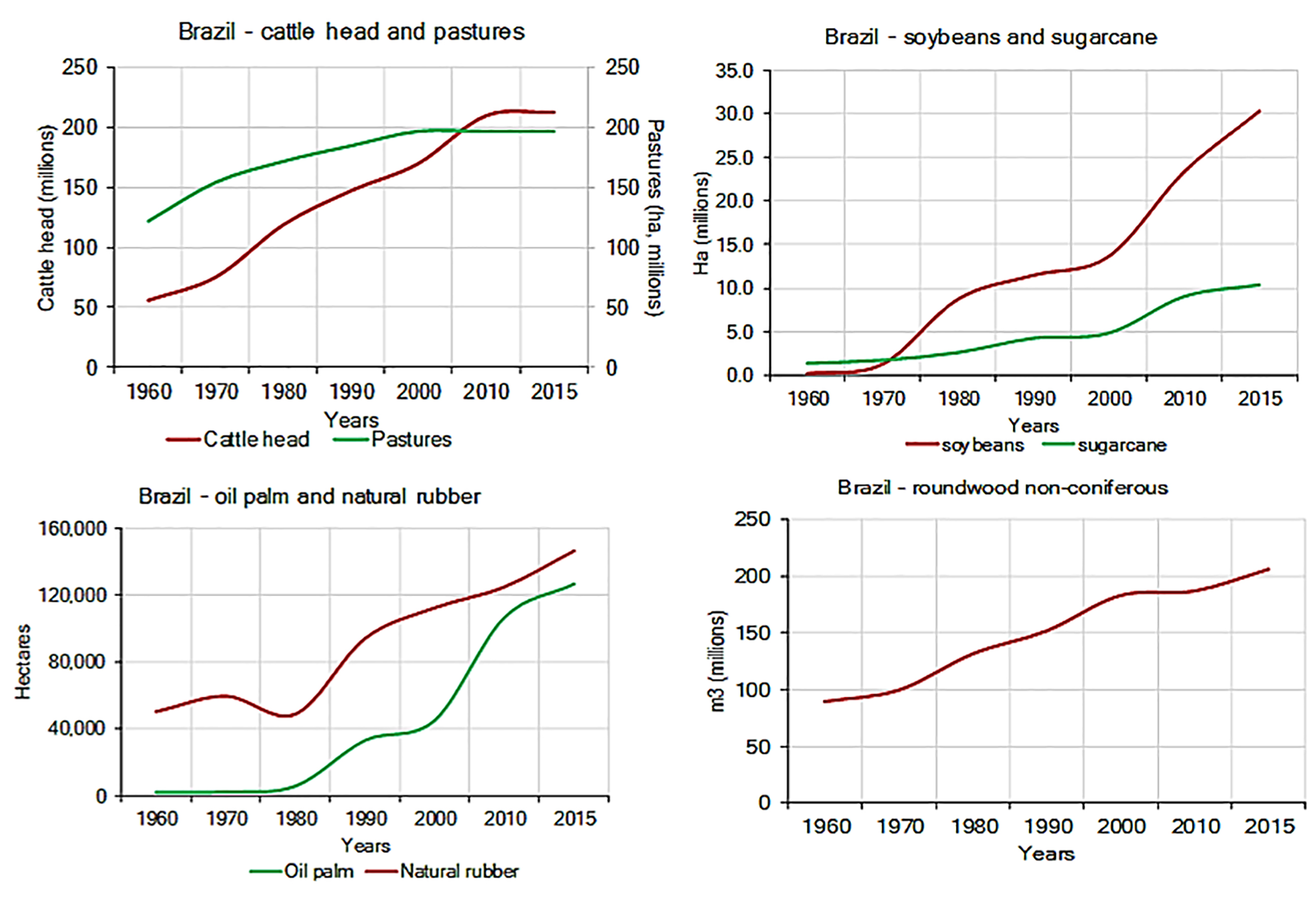

Supplement: Supplemental Information 2 — Available at http://www.fao.org/faostat/en/#data (accessed 14 February 2018; for a definition of the category Roundwood nonconiferous see http://www.fao.org/waicent/faostat/forestry/products.htm#S2; http://www.fao.org/faostat/en/#compare (crops processed). The category Roundwood-tropical has no data available in FAO data. See Text S1 for limitations of the FAO data. [file peerj-06-4869-s002.jpg]

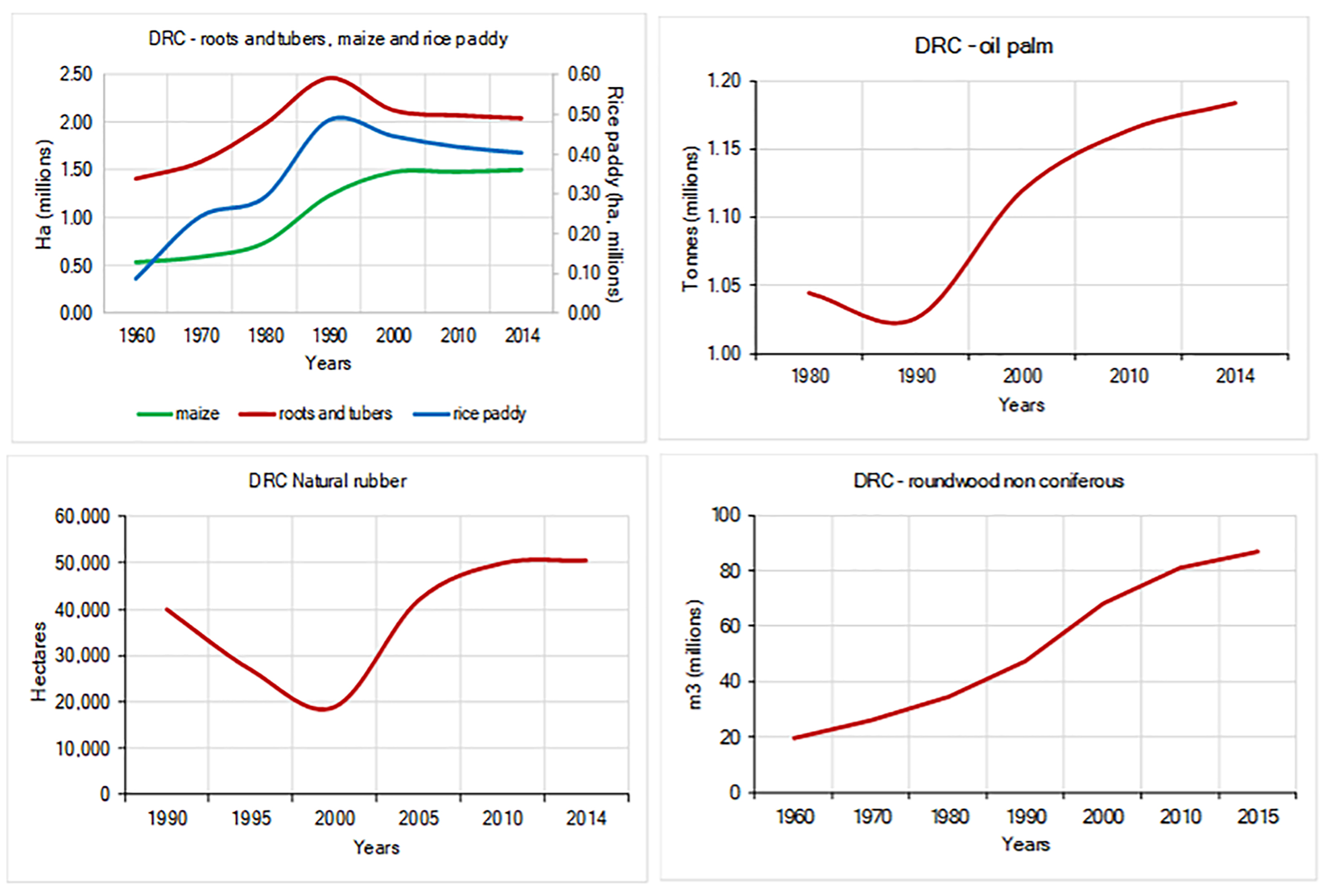

Supplement: Supplemental Information 3 — Also shown is the growth trend in the harvest of hardwoods. Available at http://www.fao.org/faostat/en/#compare (crops processed) (accessed 1 April 2017). Note: starting year may differ from one crop to another due to the lack of data available for those years in the FAO databases. See Text S1 for limitations of the FAO data. [file peerj-06-4869-s003.jpg]

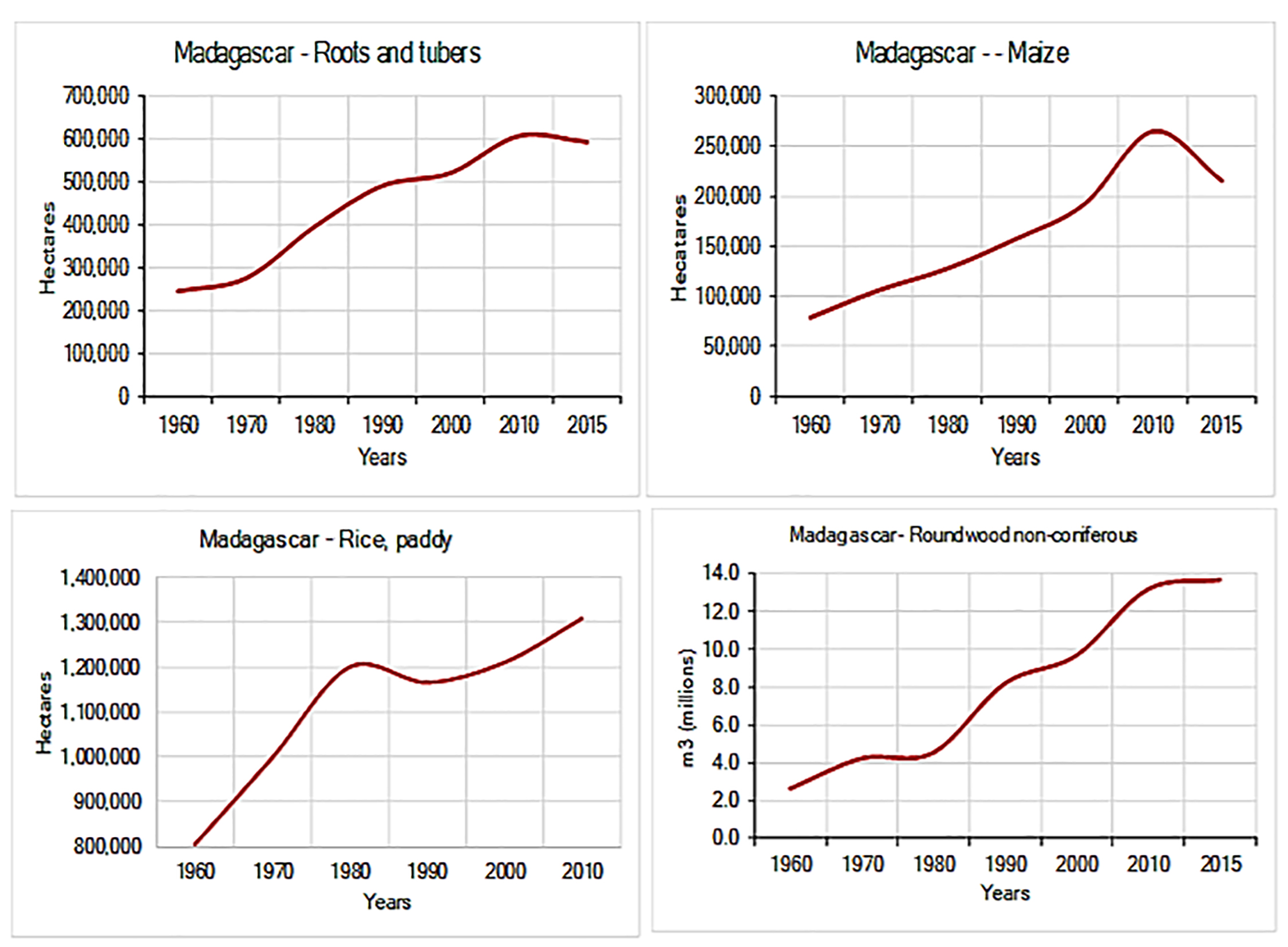

Supplement: Supplemental Information 4 — Available at http://www.fao.org/faostat/en/#compare (crops processed) (accessed 1 April 2017). See Text S1 for limitations of the FAO data. [file peerj-06-4869-s004.jpg]

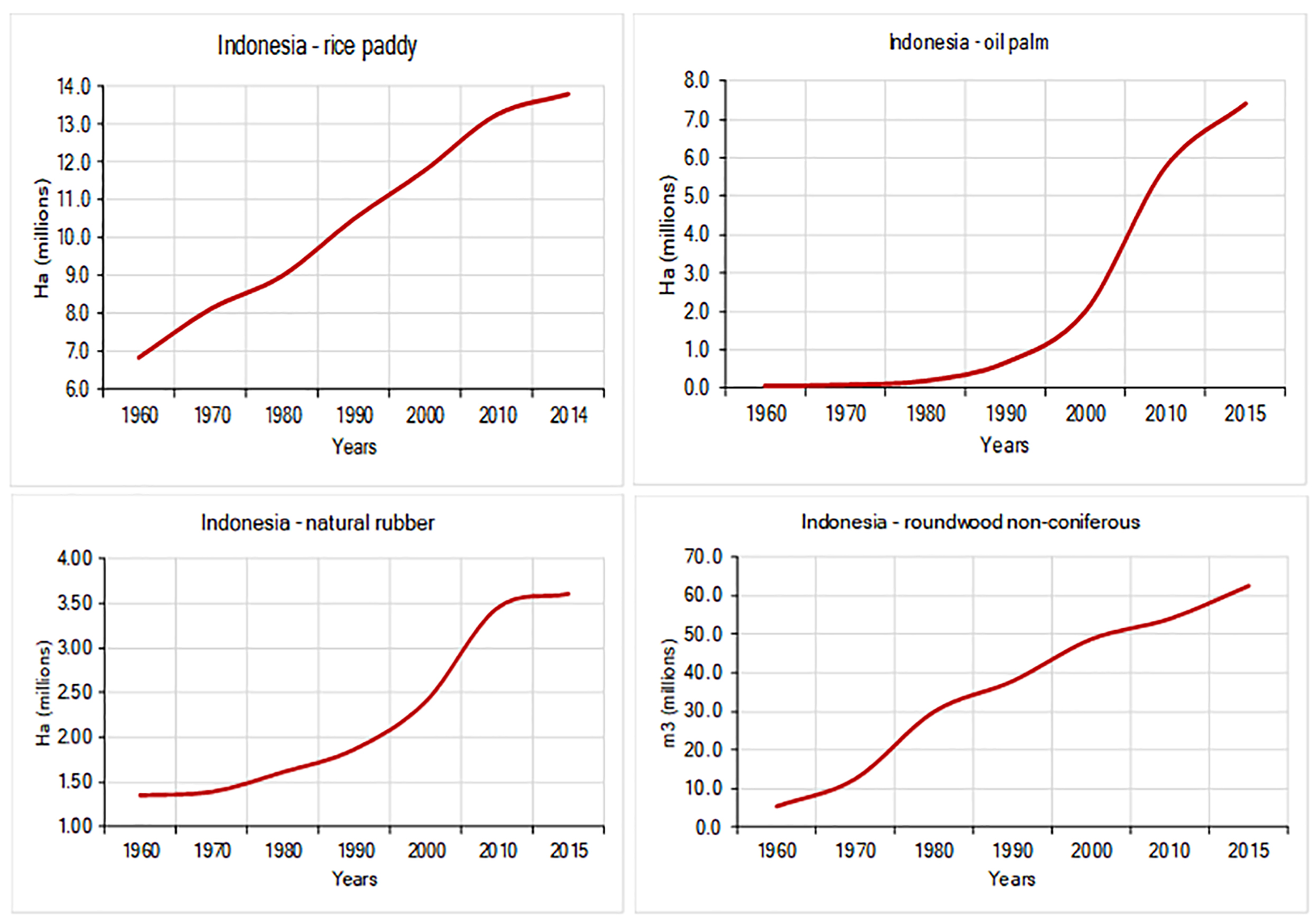

Supplement: Supplemental Information 5 — Available at http://www.fao.org/faostat/en/#compare (crops processed) (accessed 5 April 2017). See Text S1 for limitations of the FAO data. [file peerj-06-4869-s005.jpg]

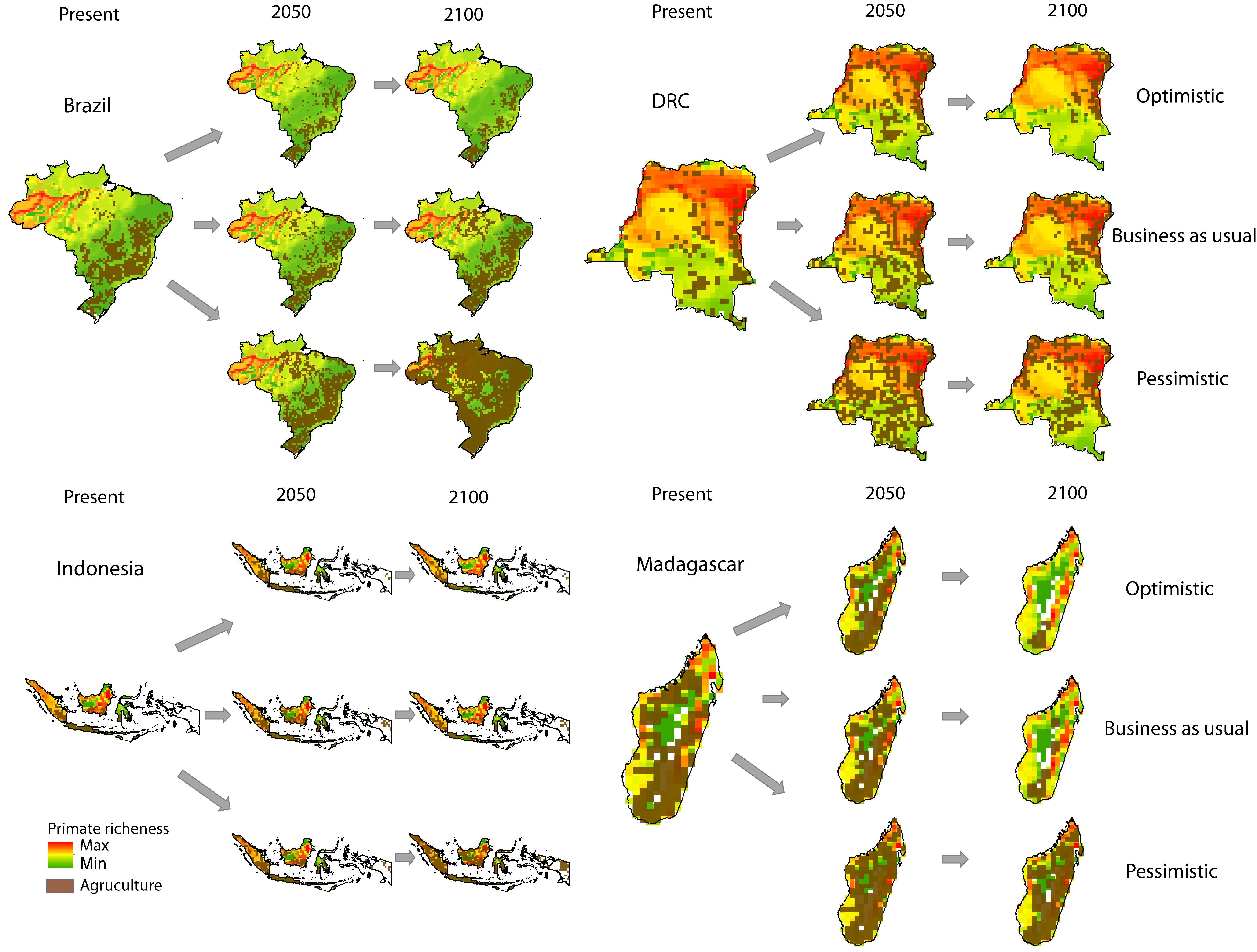

Supplement: Supplemental Information 6 — The table at the bottom shows the predicted agricultural expansion values (%) to take place by 2050 and 2100 under each of the three scenarios. Notice the spatial shift of conservation conflicts in the pessimistic models, with Madagascar and DRC reducing agricultural expansion by 2100. This is based on the expectation of the abandonment of some agricultural areas, by 2100 in DRC and Madagascar. This condition, however, may not imply an immediate benefit for primates and other species, as the areas would have been over-exploited prior to abandonment and unlikely to regenerate back to natural forest. [file peerj-06-4869-s006.jpg]

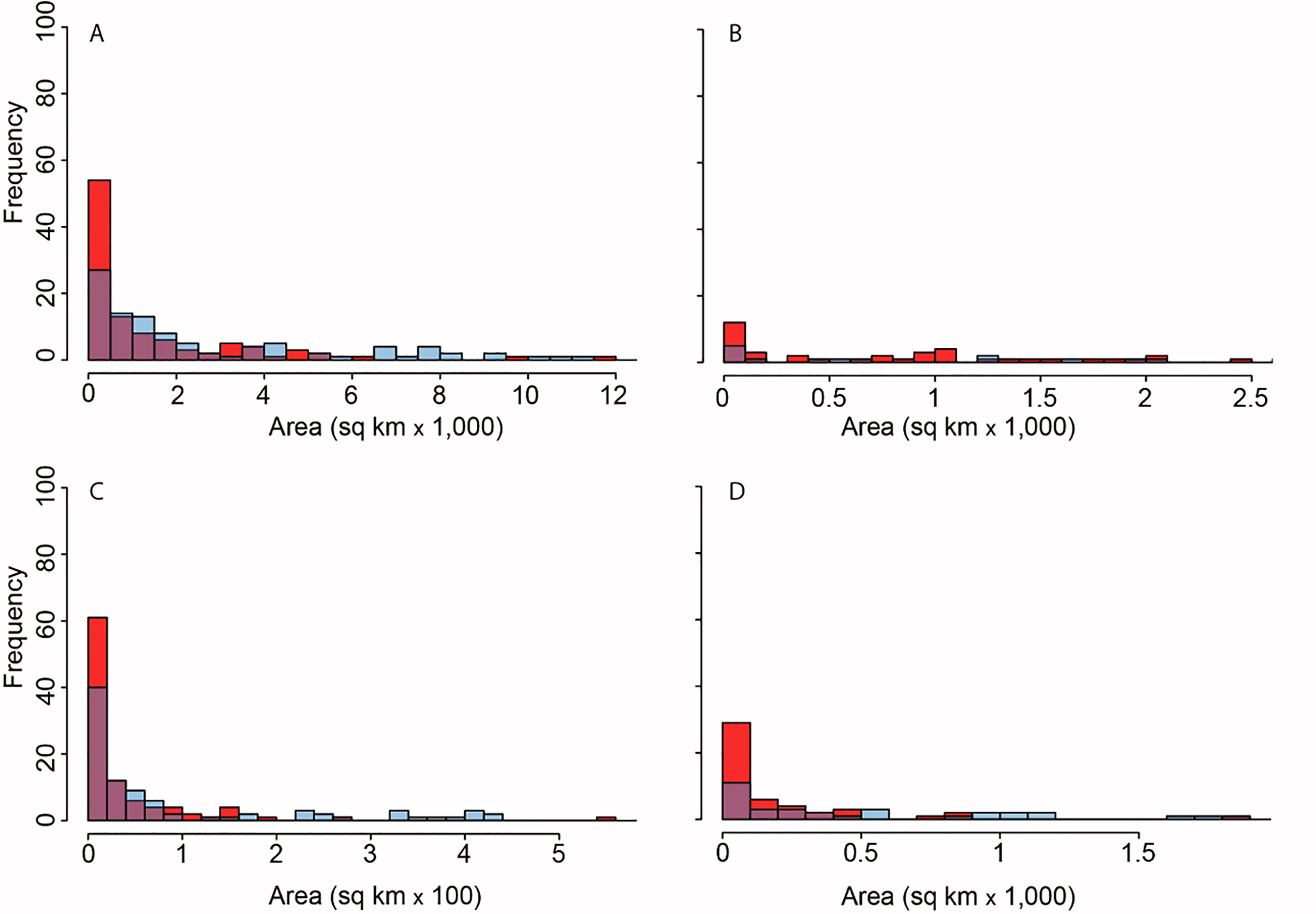

Supplement: Supplemental Information 7 — (A) Brazil, (B) DRC, (C) Madagascar and (D) Indonesia. [file peerj-06-4869-s007.jpg]
