# Supplementary material for: Primates in peril: the significance of Brazil, Madagascar, Indonesia and the Democratic Republic of the Congo for global primate conservation
Source: PeerJ. 2018 Jun 15;6:e4869. doi: 10.7717/peerj.4869 (PMC6005167; doi:10.7717/peerj.4869)
Supplement: Supplemental Information 8 — Source: IUCN, 2017 http://www.iucnredlist.org–consulted August 2017. [file peerj-06-4869-s008.docx]

|  | Mammals | Birds | Reptiles | Amphibians | Total |
| --- | --- | --- | --- | --- | --- |
| Brazil | 648 | 1,810 | 149 | 836 | 3,443 |
| DRC | 430 | 1,106 | 65 | 221 | 1,822 |
| Madagascar | 239 | 246 | 390 | 311 | 1,186 |
| Indonesia | 670 | 1,709 | 412 | 365 | 3,156 |
| Total | 1,987 | 4,871 | 1,016 | 1,733 | 9,607 |
| World | 5,466 | 11,121 | 5,473 | 6,533 | 28,593 |
| **% of World** | **36** | **44** | **19** | **27** | **34** |
