# Supplementary material for: Primates in peril: the significance of Brazil, Madagascar, Indonesia and the Democratic Republic of the Congo for global primate conservation
Source: PeerJ. 2018 Jun 15;6:e4869. doi: 10.7717/peerj.4869 (PMC6005167; doi:10.7717/peerj.4869)
Supplement: Supplemental Information 9 — Also shown is the number of species threatened and with declining populations. Source of data: IUCN, 2017 http://www.iucnredlist.org (consulted February 13th, 2018). Three families are shared by DRC and Indonesia: Lorisidae, Cercopithecidae and Hominidae. No primate species are shared by these four countries. [file peerj-06-4869-s009.docx]

|  | Brazil | Madagascar | Indonesia | DRC | Total | Global |
| --- | --- | --- | --- | --- | --- | --- |
| Species | 102 | 100 | 48 | 36 | **286** | 439 |
| Genera | 17 | 16 | 8 | 15 | 56 |  |
| Families | 5 | 5 | 5 | 4 | 16 |  |
| Species Threatened | 40 | 90 | 40 | 6 | 176 |  |
| % | 39 | 90 | 83 | 17 | **62** |  |
| Species with populations declining | 37 | 90 | 40 | 5 | 172 |  |
| % | 36 | 90 | 83 | 14 | **60** |  |
