# Supplementary material for: Primates in peril: the significance of Brazil, Madagascar, Indonesia and the Democratic Republic of the Congo for global primate conservation
Source: PeerJ. 2018 Jun 15;6:e4869. doi: 10.7717/peerj.4869 (PMC6005167; doi:10.7717/peerj.4869)
Supplement: Supplemental Information 10 — Source: Global Forest Watch (http://www.globalforestwatch.org (accessed 11 January 2018). All areas are in ha. [file peerj-06-4869-s010.docx]

| **Year** | **Brazil** | **Indonesia** | **DRC** | **Madagascar** |
| --- | --- | --- | --- | --- |
| 2001 | 2,746,361 | 745,240 | 455,435 | 86,957 |
| 2002 | 3,507,049 | 856,936 | 514,283 | 89,318 |
| 2003 | 3,248,527 | 545,405 | 274,632 | 84,248 |
| 2004 | 3,848,771 | 1,290,538 | 400,048 | 81,845 |
| 2005 | 3,486,563 | 1,184,010 | 485,218 | 93,628 |
| 2006 | 2,876,805 | 1,434,927 | 453,035 | 71,034 |
| 2007 | 2,621,820 | 1,388,842 | 462,705 | 188,580 |
| 2008 | 2,444,308 | 1,397,191 | 404,791 | 123,502 |
| 2009 | 1,817,901 | 1,946,570 | 635,134 | 136,537 |
| 2010 | 2,688,896 | 1,280,669 | 810,282 | 107,219 |
| 2011 | 1,923,364 | 1,544,772 | 429,369 | 162,323 |
| 2012 | 2,918,633 | 2,262,189 | 630,702 | 128,468 |
| 2013 | 1,945,506 | 1,140,027 | 920,509 | 299,350 |
| 2014 | 2,693,134 | 1,896,869 | 1,337,533 | 416,682 |
| 2015 | 2,222,772 | 1,748,413 | 927,932 | 301,789 |
| 2016 | 5,378,844 | 2,424,128 | 1,381,065 | 383,656 |
| Total 2001 to 2016 | 46,369,255 | 23,086,723 | 10,522,673 | 2,755,136 |
|  |  |  |  |  |
|  | |  |  |  |
