# Supplementary material for: Primates in peril: the significance of Brazil, Madagascar, Indonesia and the Democratic Republic of the Congo for global primate conservation
Source: PeerJ. 2018 Jun 15;6:e4869. doi: 10.7717/peerj.4869 (PMC6005167; doi:10.7717/peerj.4869)
Supplement: Supplemental Information 11 — Also shown is agricultural land as percent of the country’s land area. Source of data: FAOStats http://www.fao.org/faostat/en/#data (accessed 12 February 2018). Increases or decreases from year to year can be calculated by subtracting values between years. See Text S1 for limitations of the FAO data sets. [file peerj-06-4869-s011.docx]

| **Brazil** | Agricultural land km^2^ | Country’s land area km^2^ | Agricultural land as % of country land area | **Indonesia** | Agricultural land km^2^ | Country’s land area km^2^ | Agricultural land as % of country land area |
| --- | --- | --- | --- | --- | --- | --- | --- |
| 2001 | 2,634,650 | 8,515,767 | 30.94 | 2001 | 477,000 | 1,904,569 | 25.05 |
| 2002 | 2,658,680 | 8,515,767 | 31.22 | 2002 | 481,810 | 1,904,569 | 25.30 |
| 2003 | 2,684,690 | 8,515,767 | 31.53 | 2003 | 510,060 | 1,904,569 | 26.78 |
| 2004 | 2,721,320 | 8,515,767 | 31.96 | 2004 | 533,660 | 1,904,569 | 28.02 |
| 2005 | 2,724,326 | 8,515,767 | 31.99 | 2005 | 514,460 | 1,904,569 | 27.01 |
| 2006 | 2,727,840 | 8,515,767 | 32.03 | 2006 | 515,000 | 1,904,569 | 27.04 |
| 2007 | 2,716,380 | 8,515,767 | 31.90 | 2007 | 530,000 | 1,904,569 | 27.83 |
| 2008 | 2,735,000 | 8,515,767 | 32.12 | 2008 | 540,000 | 1,904,569 | 28.35 |
| 2009 | 2,735,400 | 8,515,767 | 32.12 | 2009 | 556,000 | 1,904,569 | 29.19 |
| 2010 | 2,734,630 | 8,515,767 | 32.11 | 2010 | 556,000 | 1,904,569 | 29.19 |
| 2011 | 2,753,730 | 8,515,767 | 32.34 | 2011 | 565,000 | 1,904,569 | 29.67 |
| 2012 | 2,756,070 | 8,515,767 | 32.36 | 2012 | 565,000 | 1,904,569 | 29.67 |
| 2013 | 2,788,081 | 8,515,767 | 32.74 | 2013 | 570,000 | 1,904,569 | 29.93 |
| 2014 | 2,825,890 | 8,515,767 | 33.18 | 2014 | 570,000 | 1,904,569 | 29.93 |
| 2015 | 2,825,890 | 8,515,767 | 33.18 | 2015 | 570,000 | 1,904,569 | 29.93 |
| Growth between 2001 and 2015 **2.25%** | | | | Growth between 2001 and 2015 **4.88%** | | | |
|  | | | |  | | | |

| **Madagascar** | Agricultural land km^2^ | Country’s land area km^2^ | Agricultural land as % of country land area | **DRC** | Agricultural land km^2^ | Country’s land area km^2^ | Agricultural land as % of country land area |
| --- | --- | --- | --- | --- | --- | --- | --- |
| 2001 | 408,430 | 587,041 | 69.57 | 2001 | 255,500 | 2,344,858 | 10.90 |
| 2002 | 408,430 | 587,041 | 69.57 | 2002 | 255,500 | 2,344,858 | 10.90 |
| 2003 | 408,430 | 587,041 | 69.57 | 2003 | 255,500 | 2,344,858 | 10.90 |
| 2004 | 408,430 | 587,041 | 69.57 | 2004 | 255,500 | 2,344,858 | 10.90 |
| 2005 | 408,930 | 587,041 | 69.66 | 2005 | 255,500 | 2,344,858 | 10.90 |
| 2006 | 408,930 | 587,041 | 69.66 | 2006 | 255,860 | 2,344,858 | 10.91 |
| 2007 | 408,930 | 587,041 | 69.66 | 2007 | 256,500 | 2,344,858 | 10.94 |
| 2008 | 410,950 | 587,041 | 70.00 | 2008 | 257,000 | 2,344,858 | 10.96 |
| 2009 | 413,950 | 587,041 | 70.51 | 2009 | 257,450 | 2,344,858 | 10.98 |
| 2010 | 413,950 | 587,041 | 70.51 | 2010 | 257,650 | 2,344,858 | 10.99 |
| 2011 | 414,050 | 587,041 | 70.53 | 2011 | 260,400 | 2,344,858 | 11.11 |
| 2012 | 414,150 | 587,041 | 70.55 | 2012 | 262,000 | 2,344,858 | 11.17 |
| 2013 | 414,150 | 587,041 | 70.55 | 2013 | 262,000 | 2,344,858 | 11.17 |
| 2014 | 414,150 | 587,041 | 70.55 | 2014 | 262,000 | 2,344,858 | 11.17 |
| 2015 | 414,150 | 587,041 | 70.55 | 2015 | 262,000 | 2,344,858 | 11.17 |
| Growth between 2001 and 2015 **0.98%** | | | | Growth between 2001 and 2015 **0.28%** | | | |
|  | | | |  | | | |
