# Supplementary material for: Primates in peril: the significance of Brazil, Madagascar, Indonesia and the Democratic Republic of the Congo for global primate conservation
Source: PeerJ. 2018 Jun 15;6:e4869. doi: 10.7717/peerj.4869 (PMC6005167; doi:10.7717/peerj.4869)
Supplement: Supplemental Information 12 — Source HDI: http://hdr.undp.org/en/countries/profiles/COD (accessed 5 February 2018) Source GDPPC: http://data.worldbank.org/indicator/NY.GDP.PCAP.CD?contextual=max&locations=BR&year_high_desc=false; http://data.worldbank.org/indicator/NY.GDP.PCAP.CD (accessed 5 February 2018). [file peerj-06-4869-s012.docx]

|  | 12-APRIL-2017 |  |  |
| --- | --- | --- | --- |
| Rank |  | GDP per capita (US$) | HDI (0 least-1.0 most) |
| 1 | Luxembourg | $101,450.0 | 0.897 |
| 2 | Norway | $89,492.8 | 0.949 |
| 3 | Switzerland | $75,531.5 | 0.939 |
| 4 | Qatar | $74,686.6 | 0.856 |
| 5 | Ireland | $65,292.4 | 0.923 |
| 6 | Denmark | $58,098.3 | 0.925 |
| 7 | Sweden | $55,186.0 | 0.913 |
| 8 | Australia | $54,708.2 | 0.939 |
| 9 | US | $51,638.1 | 0.920 |
| 10 | Netherlands | $51,268.5 | 0.924 |
| 11 | Canada | $50,000.6 | 0.920 |
| 12 | Austria | $47,754.6 | 0.893 |
| 13 | Japan | $47,150.4 | 0.903 |
| 14 | Iceland | $45,411.0 | 0.921 |
| 15 | Germany | $45,408.3 | 0.926 |
| 16 | Findland | $45,132.8 | 0.895 |
| 17 | Belgium | $45,036.1 | 0.896 |
| 18 | United Kingdom | $43,876.0 | 0.909 |
| 19 | France | $41,533.9 | 0.897 |
| 20 | New Zealand | $36,801.4 | 0.915 |
| 21 | Italy | $33,849.4 | 0.887 |
| 22 | Spain | $30,587.6 | 0.884 |
| 23 | Korea (Republic of) | $25,022.8 | 0.901 |
| 24 | Greece | $22,573.4 | 0.866 |
| 25 | Czetch Republic | $17,548.3 | 0.878 |
|  |  |  |  |
| Rank |  |  |  |
| 86 | Brazil | $8,538.6 | 0.740 |
| 137 | Indonesia | $3,346.5 | 0.689 |
| 202 | Madagascar | $401.8 | 0.512 |
| 204 | DRC | $456.1 | 0.435 |
