# Supplementary material for: Primates in peril: the significance of Brazil, Madagascar, Indonesia and the Democratic Republic of the Congo for global primate conservation
Source: PeerJ. 2018 Jun 15;6:e4869. doi: 10.7717/peerj.4869 (PMC6005167; doi:10.7717/peerj.4869)
Supplement: Supplemental Information 13 [file peerj-06-4869-s013.docx]

**Estrada et al.**

**Supplementary text (Text S1)**

**Text S1**

**Survey methodology**

List of agencies consulted for data sets used to profile time series of parameters of interest for each of the four countries under investigation. The list includes the URLs of the sections of the agencies’ websites where origin, accuracy and other aspects of the nature of the data sets are found.

**FAO** Food and Agriculture Organization of the UN

**http://www.fao.org/faostat/en/#definitions**

**http://www.fao.org/statistics/en**

**The World Bank**

**http://ieg.worldbankgroup.org/methodology**

**IUCN** International Union for the Conservation of Nature Red List

**http://www.iucnredlist.org/about/overview**

**GFW** Global Forest Watch

**https://www.globalforestwatch.org/**

**CITES**

**https://trade.cites.org/;**

**https://trade.cites.org/cites_trade_guidelines/en-CITES_Trade_Database_Guide.pdf**

**ITC** International Trade Centre

**http://www.intracen.org***/*

**http://www.intracen.org/itc/market-info-tools/market-analysis-tools/**

**Protected Planet (UNEP-WCMC)**

**https://www.protectedplanet.net/c/about**

**https://www.protectedplanet.net/c/terms-and-conditions**

**IMAGE** Integrated Model to Assess the Global Environment

**http://themasites.pbl.nl/models/image/index.php/Agricultural_economy**

**http://themasites.pbl.nl/models/image/index.php/Agricultural_economy/Data_uncertainties_limitations**

**UN Development Program Human Development Index (HDI)**

**http://hdr.undp.org/en/content/human-development-index-hdi**

[**http://hdr.undp.org/en/statistics/understanding**](http://hdr.undp.org/en/statistics/understanding)

**GPI (Global Peace Index)** **of** **the Institute for Economics and Peace** **<http://economicsandpeace.org/>.**

**http://economicsandpeace.org/research/#risk; http://economicsandpeace.org/about/**

**The 2016 Transparency International Corruption Perceptions Index (CPI)**

https://www.transparency.org/news/feature/corruption_perceptions_index_2017

https://www.transparency.org/news/feature/corruption_perceptions_index_2017#resources; https://www.transparency.org/whoweare/accountability

**Richness of primate taxa and IUCN conservation and population status**

Brazil, Madagascar, DRC and Indonesia are important reservoirs of the world´s biodiversity, being considered megadiverse countries (*Mittermeier, Robles Gil & Mittermeier, 1997*). For example, together they account for 36% of the world’s terrestrial mammals (n = 5,466), 44% of all birds (n = 11,121), 19% of all reptiles (n = 5473) and 27% of all amphibians (n = 6533) (Table S2). Brazil and DRC account for 64% and 60% of the Amazon (7.5 million km^2^) and Congo (3.5 million km^2^) basins, respectively (*Ernst et al., 2013*; *Rittera et al., 2017*; Fig. 1). Madagascar is the largest African island and the fourth-largest island in the world, after Greenland, New Guinea, and Borneo. It has been isolated for about 80 million years and contains the only remaining radiation of lemurs (*Zimkus et al., 2017*). Indonesia, the world's largest island country, is composed of more than seventeen thousand islands (*CIA, 2017*). Each of the four countries is characterized by a unique biogeographical history that has nurtured a highly successful radiation of endemic and more widely distributed primate taxa.

**Expansion of agricultural land**

While forest loss and agricultural expansion are common patterns in all four countries, each country differs in its type of agricultural production due to domestic and global market demands. Below we document some of these between country differences. It is worth noting that the quality of the data provided to the FAO is not the same for all countries. DRC in particular has serious infrastructure issues, including the lack of widespread power and transport lines (apart from rivers) throughout much of the country and a poorly-resourced civil service, limiting the country’s capacity to measure and report metrics that cannot be analysed using remote sensing, such as agricultural land in use, and productivity of any given crop. The World Bank’s Statistical Capacity Score (a metric reflecting a nation’s ability to collect, analyze, and disseminate high-quality data about its population and economy) reflects the likely precisión of statistics reported to the FAO. Scores are out of 100 (greatest precision) and are: DRC 46.7, Madagascar 56.7, Brazil 74.4 and Indonesia 86.7. This means that FAO data quality for DRC and Madagascar is poor. *http://databank.worldbank.org/data/reports.aspx?source=Statistical-capacity-indicators*

**Brazil**

The cattle population in Brazil grew from 169 million head in 2000 to 212 million in 2015 (larger than Brazil’ human population) with concomitant increases in pasture (*ABIEC BRAZIL, 2016*). Brazil has greatly expanded its production of soybeans (*Glycin max*), sugarcane (*Saccharum* spp.), oil palm (*Elaeis guineensis*), natural rubber (*Hevea brasiliensis*), and timber in recent decades, at the expense of primate habitats ([http://www.fao.org/](http://www.fao.org/faostat/en/#data)consulted November 2017; Fig. S2). Soybean crops more than doubled from 13.6 million ha in 2000 to 30.3 million ha in 2015. Sugarcane expanded in already established pastures from 4.8 million ha in 2000 to 10.4 million ha by 2015. Oil palm and natural rubber plantations have rapidly expanded as well. The former, from 45,000 ha in 2000 to 126,559 ha in 2015. The latter, from 112,396 ha to 146,552 ha over the same 15-year period. Nonconiferous roundwood extraction in Brazil has grown from 183 million m^3^ in 2000 to 205 million m^3^ in 2015 ([*http://www.fao.org*/](http://www.fao.org/faostat/en/#data)consulted November 2017; Fig. S2 for definition of FAO category).

**DRC**

In DRC the rural complex (*sensu* *Molinaro et al, 2017*) covers about 13.1% of the country’s land surface. This rural complex includes roads and villages (14% of the complex, and thus uncultivable), active and fallow fields, secondary forest and primary forest (approximately 11% of the complex); 5% of the complex are clearings and 10% are active fields; the rest (60%) is fallow land (*Molinario et al., 2017)*. The majority (76%) of the rural complex in DRC is already part of the roughly 18-year cycle of shifting cultivation, and therefore most of the forest lost within the complex represents the cyclical removal of secondary forest (*Molinario et al., 2015*; *Molinario et al., 2017*). The net annual increase of expansion into primary forest outside of the rural complex, often into protected areas is very low – about 1% of the total land area annually (*Molinario et al. 2015*). The loss of primary forest is of greatest concern for primates in DRC.

Bushmeat extraction in central African forests was already six times higher than the sustainable rate by 1999 (*Bennett et al., 2002*), and primates are one of the guilds least able to withstand any but the lightest hunting pressure (*Robinson & Bennett 2000*). Few of the larger-bodied primates in DRC are now likely to inhabit the rural complex, which has been heavily hunted in the last few decades (*Ziegler et al., 2016*).

Most land clearing in the rural complex in DRC is land that has already been cleared in the last two decades. Commercial plantations (such as rubber and oil palm cover 2% of the rural complex (*Molinario et al. 2017*) cover approximately 2,580 km^2^. Of these concessions (N=150), only one, which covers 464 km^2^ (18% of all the industrial agricultural concessions), has its center in an IFL (Intact Forest Landscape). After independence, and especially between 1996-2002, during a period of extreme political instability and civil war in DRC, commercial agricultural production fell sharply, and virtually ceased. Since then, and especially in the last decade (2006 onwards), the old pre-independence plantations have been rehabilitated on land that was cleared in colonial times or up to the mid-1990s. A handful of companies own the industrial plantations, and produce about 50,000 tons of palm oil annually. Another 50,000 tons are produced by village plantations, and the remainder – 200,000 tons – are collected from old trees in the rural complex, including from abandoned plantations (Semroc *et al.* 2015). Almost all production is consumed in-country. Given the predicted growth in the human population, DRC will need to plant an additional 1,600 km^2^ by 2030 (*Semroc et al. 201*5). Although FAO suggests that between 2000 and 2015, 12 million ha were deforested to produce these crops (Fig. S3), most of this involved the rehabilitation of existing palm and rubber concessions, which had already been deforested when they were established at the beginning of the 1900s. Between 2000 and 2015 natural rubber plantations in DRC more than doubled from 19,000 ha to 50,600 ha and the production of oil palm fruit increased from 1.12 million metric tons in 2000 to 1.18 million metric tons in 2015 (Fig. S3). These areas are all in sites close to human settlements (*GFW, 2018*) and primates are thus likely to have been hunted out several decades ago (*Ziegler et al., 2016*).

The humid forest area of the country is primarily a forest-based farming system where the principal crops are manioc (cassava; *Manihot esculenta*) and plantains (*Musa* spp.) in forested areas, and to a smaller degree rice and maize in both forested and savannah areas (*Dixon et al., 2001*).

**Madagascar**

In Madagascar, the cultivation of roots. tubers, maize, and especially rice mostly for internal consumption and export, is an important driver of losses of native vegetation. The cultivation of these crops increased from 1.9 million ha in 2000 to 2.2 million ha in 2010. More recently the harvesting of roundwood for export has accelerated the pace of deforestation, and forest degradation and extraction grew from 9.7 million m^3^ in 2000 to 13.7 million m^3^ in 2015 (Fig. S4). In Madagascar, rapid agricultural expansion and associated habitat loss and degradation combined with logging and hunting have resulted in population declines in 90% of lemur species (e.g. Verreaux's Sifaka, *Propithecus verreauxi*) (Fig. 1).

**Indonesia**

Rice cultivation in Indonesia expanded from 8.3 million ha in 2000 to approximately 14 million ha in 2015 (Fig. S5). Global market demands have greatly accelerated the conversion of forested land to oil palm and natural rubber plantations. Oil palm plantations in Indonesia expanded from 2 million ha in 2000 to 7.4 million ha in 2015. Natural rubber plantations have increased from 2.4 million ha in 2000 to 3.6 million ha in 2015. Industrial roundwood extraction increased from 48.6 million m^3^ in 2000 to 62.4 million m^3^ in 2015 (Fig. S5). The rapid conversion of forested land to oil palm and rubber plantations in Indonesia has resulted in the loss of habitat (including protected areas) for critical many primates (e.g, orangutans, *Pongo* spp., as well as other rare primates with which they share their habitats) (*Ahrends et al., 2015*; *Warren-Thomas et al., 2015*; *Nantha & Tisdell, 2009*; *Nater et al., 2017*; *Struebig et al., 2015*); all three orangutan species are now classified as Critically Endangered (*IUCN 2017*).

**Modeling agricultural expansion and primate range contraction in the 21^st^ century**

We present a country by country summary of the main findings of the analyses of spatial conflict between primate species and predicted agricultural expansion during the 21^st^ century. Species distributions were obtained from the IUCN range maps (*IUCN*, 2017). Agricultural expansion is derived from the IMAGE database and represents the predicted presence (irrespective of the intensity) of agricultural production at each grid cell (0.5° of spatial resolution; see *Dobrovolski et al. 2014*). Therefore, agriculture should be viewed as a descriptor of conflict between primate occurrence and agriculture.

**Brazil**

Data on primates from Brazil included 104 species. Currently, the average spatial overlap between agriculture and individual species ranges is 10.3%, ranging from 0.0% to 60% (the greatest overlap is for the northern muriqui. The optimistic scenario is predicted to alleviate this conflict, resulting in an average overlap of 6% by 2050, ranging from 0.0% to 50% in *Leontopithecus chrysopygus*, and 4% by 2100 (0.0 to 40% in the optimistic scenario). The pessimistic scenario points to an average spatial overlap of 19.6% (0.0 – 75%) by 2050 and 79% by 2100. According to this pessimistic scenario, by the end of this century, 15 species (14.4%) will face conflict with agriculture in more than 80% of their geographical distribution. Considering a business-as-usual scenario, spatial conflict will affect, on average, 11.5% (0.0 – 68%) and 18% (0.0 – 79%) of the range of the species by 2050 and 2100, respectively.

**DRC**

Data for DRC included 34 species. Current spatial overlap, on average, is 12% of the species range (varying from 0% to 41%). Under an optimistic scenario, spatial conflict will vary from 10.7% to 18% between 2050 and 2100 but is expected to reach as high as 54% (for both periods). Presently, as well as in the optimistic and business-as-usual scenarios for 2050 the species with the greatest overlap with agriculture is *Galago matschiei*. In the business-as-usual scenario, spatial overlap with agriculture will affect 17.5% to 24.3% of the species ranges by 2050 and 2100, respectively. Higher overlap values are expected for *Gorilla beringei* (51.3%) and *Galago matschiei* (44%). In a worst-case scenario, average spatial overlap is predicted to be 34% by 2050, and to average 23.3% by 2100, varying between 0% and 53% (for *Gorilla beringei*). Note that, by 2100, the average spatial conflict with agriculture will be lower for most species. This is because agriculture is predicted to shift geographically, including abandonment of some areas.

**Indonesia**

Data for Indonesia included 48 species. From these, 13 species were not evaluated properly as they occur in very small islands lacking accurate information on agriculture at the spatial resolution adopted here (i.e., 0.5°). For those species properly evaluated, we found that currently, 15.5% of their geographic ranges are under spatial conflict with agriculture. In the optimistic scenario, this average overlap is 21% by 2050 and 18% by 2100. In the business-as-usual scenario, spatial conflict will, on average, reach 25% to 21% by 2050 and 2100, respectively. In the pessimistic scenario, we predict spatial overlap of 24.5% to 39.7% by 2050 and 2100, respectively. The maximum overlap with agriculture is found for *Macaca maura*, totaling 81.4% of its range in all scenarios and periods. *Presbytis comate* also faces strong spatial conflict with agricultural expansion (about 80% of its range, regardless of the scenario and period).

**Madagascar**

Data for Madagascar included 97 species. From these, two species are already under strong spatial conflict with agriculture, with their entire range coinciding with areas of agricultural use. These species are the endangered *Lepilemur wrightae* and the critically endangered *Hapalemur alaotrensis*. Current average spatial overlap between agriculture and species’ ranges is 33%. By 2050, this overlap is estimated at 36% for the optimistic model, 31.7% for the business-as-usual model, and 47% for the pessimistic model. By 2100, in the optimistic and business-as-usual scenarios, the spatial extent of agriculture is predicted to decrease substantially in Madagascar, reducing the conflict to 2.7% and 4.5%, respectively. In the pessimistic scenario, the overlap reaches 40% by 2100.

**Logging**

Unlike the other countries of the region, DRC has only a relatively small proportion (8%) of forest assigned as logging concessions. A moratorium on all new logging concessions was ratified into law in 2002 (*Debroux et al., 2007*) and in 2009, 60% of all existing concession contracts were cancelled due to legal issues (*ITTO, 2009*). In DRC, logging is practiced selectively (a few trees are extracted per hectare), removing 10-20% of the canopy (2-10 trees/ha). Recent work in the neighboring Republic of Congo has shown that great apes can persist in such forests if hunting is strictly controlled *(Morgan et al., 2017*). Currently, few logging concessions are guarded or protected in DRC, and guards are unlikely to be employed in logging concessions that are not certified by the Forest Stewardship Council (FSC). At present no concession in DRC is FSC-certified (*https://info.fsc.org/certificate.php#result*).

**Mining**

**DRC**

When mining concessions are opened in remote forests in DRC, access along new road networks is greatly increased. This access facilitates bushmeat hunting. The type of mining practiced in DRC tends to attract thousands of people hoping to strike it rich. These people have greater purchasing power than a rural farmer and can afford to pay high prices for bushmeat, thus increasing the incentive to hunters of harvesting more animals from the forest than they require for their own families’ protein needs. It is important to note, that a mining *prospection permit* is not the same as a mine: nevertheless, a prospection permit carries the risk that a mine will be opened, as ore extraction becomes more profitable over time. Of the existing 1,249 mining prospection permits in DRC, 952 (76%) have their centers in the rural complex (areas that have been in the cycle of slash-and-burn agriculture for at least 18 years). Permits in the rural complex cover 143,316 km^2^, which is 78% of the total permitted area.

**International commercial trade of commodities and primate habitat loss**

Current trends in the conversion of native vegetation to agricultural fields and wood extraction are the result of large-scale market demands by upper and middle-income nations (*World Bank, 2017*) and the actions of primate harboring countries to develop their own economies and improve the standard of living of their growing human populations. For example, in 2015 Brazil had the second largest cattle inventory in the world (*ca* 212 million cattle head), after India (*ca* 298 million cattle head) (*ABIEC BRAZIL, 2016*). In 2016, frozen beef was exported by Brazil to 38 countries, with China importing 40% of the total (see below for sources). Note that the growth of pastures is strongly associated with the growth of the cattle industry (Fig. S1). After the USA, Brazil is the largest producer of soybeans (<http://www.globalsoybeanproduction.com/> consulted May 2017) in the world. Brazil´s estimated production of soybeans in 2016 was a record *ca* 111 million metric tons (*USDA, 2017*). In 2016, Brazil exported soybeans to 27 countries, with 74% of the crop exported to China (see below for sources). Much of land devoted to soybean cultivation in Brazil is in areas that naturally contained forests and other vegetation types as well as high primate diversity.

In 2016, DRC exported minerals to eight countries but 96% of these exports went to China (60%), Malaysia (15%), Germany (11%) and India (9%). Another DRC export is natural rubber. In 2016, exports of natural rubber went to five countries, Malaysia (65%), France (16%), Romania (14%), Spain (4%) and Germany (1%). In 2016, DRC exported tropical wood to 18 countries with 84% of the share taken by China (47%), France (20%), Portugal (9%) and Belgium (8%) (see below for the source of these data). At the time of writing (March 10, 2018) DRC has passed a new law which raises the tax to be paid by mining companies from 2% to 10%, a move opposed by many companies who say it will render their operations unprofitable.

Madagascar´s top exports are minerals. In 2016, minerals were exported to eight countries but three of these countries accounted for 90% of exports (Canada 34%, China 29%, and the USA 27%). Coffee, tea, and spices also are exported by Madagascar to 29 countries, with 67% of the share taken by France (22%), the USA (21%), Germany (15%) and India (9%). Edible vegetables, roots and tubers are exported by Madagascar to 39 countries with India (36%), France (10%), and Pakistan (5%) accounting for the largest share (see source below for individual data sets).

Indonesia´s cultivation of rice is rapidly expanding, and rice exports provide important revenues to the country. In 2016, Indonesia exported rice to 11 countries in Asia, North America, and Europe and Australia, with four countries purchasing 80% of the share (India 27.2%, Thailand 22.1%, Singapore 20.7% and the Philippines 10.6%). Natural rubber, another growing and important export of Indonesia, was sent to 45 countries in 2016, with four countries importing 59% of production (USA 22%, Japan 16%, China 12%, and India 9%) (see below for the source of these data). Indonesia also exported tropical wood to 47 countries in 2016, with 51% of their production imported by three countries (China 21%, Japan 21%, and the USA 9%) (see below for the sources of this information.

General international trade maps and trade maps for selected exports for Brazil, the Democratic Republic of the Congo, Madagascar and Indonesia can be found in: International Trade Centre, 2016; http://www.intracen.org/itc/market-info-tools/trade-statistics/

Sources consulted for each country in May 2017.

**Legal and illegal primate trade**

**Legal Trade**

The data presented in Table 3 are from the CITES trade database on all international trade entries for each of the four countries for the period 2006-2016 (data from 2016 were incomplete but were included as to be as current as possible). All transactions involving primates, irrespective of the purpose of the trade, were included. For analysis, we focussed on (1) live animals, (2) bodies, skeletons and skins, and (3) specimens, as reported in the CITES trade database. Specimens were principally exported for medical or scientific purposes, and partially exported for commercial proposes. We excluded entries that were reported in g, kg, l, or ml. For each country, we calculated the proportion of trade that comprised wild-caught individuals (see *Fialho, Ludwig, Valença-Montenegro, 2016* for an analysis of the legal trade of Neotropical Primate originated in South American countries from 1977 to 2013).

**Brazil: live trade**

Legal international trade in night monkeys *Aotus* spp from Brazil appears to be very limited (Svensson et al. 2016) but illegal cross-border trade in the Brazil-Colombia-Peru tri-border area continues to flourish (*Maldonado et al., 2009*). One trader in Brazil indicated illegally exporting ~2000 night monkeys, most likely *A. nancymaai* or *A. vociferans*, over an 18-year period, and six such traders were identified by Maldonado et al. (2009) to be working close to the Peru-Colombia border. *Do Nascimento, Schiavetti & Montaño* (*2013*) reported that 15 capuchin monkeys *Sapajus* spp in rescue centers and 105 in zoos in the State of Bahia, Brazil, were derived from the illegal trade, and noted that the demand for primates as pets in Brazil, was one of the drivers. *Da Silva et al.,* (*2016*) modelled the effect of hunting to meet the demand for pets on populations of the yellow-breasted capuchin *S. xanthosternos*, an endemic species, showing that even a small offtake can have an important negative impact on species survival. However, they did not provide the number of capuchins that were extracted from the wild. Overall it seems that at present, the live trade in primates in Brazil is limited in terms of number of individuals and the number of species that are negatively affected.

In the state of Rio de Janeiro, Brazil, from 2003-to 2010 approximately 457 primate specimens were delivered to the CETAS (an animal rescue and rehabilitation center) while another 438 were recorded in citizen phone complaints that denouced illegal pets (Linha Verde). Most of the records were of the genera *Callithrix* (363 and 415 records) and *Cebus/Sapajus* (86 and 17 records) respectively although other genera (*Leontopithecus, Callicebus, Lagothrix* and *Alouatta*) also were recorded (*Oliveira & Grelle 2012*). Although the impact of such numbers in the populations of these species may be not significant, the impact of released animals outside their native range may threaten native species, by hybridization, as the case of the Buffy-tufted-ear marmoset, *Callithrix aurita* in Rio de Janeiro (*Oliveira & Grelle 2012*).

**Brazil: medicinal or meat**

Ferreira et al. (2013) surveyed animal markets throughout Brazil and found that primates made up only 1 of 131 species offered for sale. Capuchin penises, bones and fat were the parts listed as being used for medicinal purposes. *Van Vliet et al.,* (*2014*), working in the Brazil-Colombia-Peru tri-border area noted that, in terms of individuals, brown woolly monkeys, *Lagothrix lagothricha,* made up 10% (31/311) of the 16 animal species that Brazilian hunters caught in the area. These hunters sold 96% of the biomass they extracted and only 4% was used for their own consumption. In terms of the number of individuals, the brown woolly monkey was ranked 9th out of 14 species that were offered for sale in two Brazilian wildlife markets (*Van Vliet et al., 2014*).

**DRC: live trade**

Trade in live primates in DRC seems to be much less of a conservation challenge than the trade in primates for bushmeat. However, often the trade in live primates, especially infants and young juveniles, is directly linked to the bushmeat trade. *Svensson et al.,* (*2015*) reported on the trade in lorisiforms in Congo DRC and noted that pottos (*Perodicticus ibeanus)* are traded in small numbers as pets, and this same species also was traded as bushmeat (*Musibono et al., 2010*). *Hicks et al.,* (*2010*) reported on the trade of 42 chimpanzees as pets in northern DRC over an 18-month period. *Andre et al.* (*2008*) reported that 42 bonobos that had been kept as pets arrived at a sanctuary (either as donations or as part of a confiscation) over a 12-year period.

**DRC: medicinal and bushmeat trade**

*Hicks et al.* (*2010*) found 34 chimpanzee carcasses, killed for the purposes of bushmeat trade over an 18-month period in northern DRC. Primates were extracted at a rate of 26 tons a year (the equivalent of ~5,000 individuals) in two districts in the Ituri forest, partially to meet the demand for the commercial bushmeat trade (*Wilkie et al., 1998*). In this region, primates were the most commonly hunted (and traded) taxon after duikers (*Nasi et al., 2011*). Van Vliet et al. (*2012*) monitored a bushmeat market in Kisangani over a 12-month period (131 one-day visits) and found 8,228 monkeys of the genera *Chlorocebus* and/or *Cercocebus* for sale, in addition to 139 baboons (*Papio spp*), 103 chimpanzees and 17 bonobos. These numbers translate to a turnover of 65 primates / day. *Dupain et al. (2012)* recorded 4,620 primate carcasses in the market of Basankusu, a rate of 17 carcasses per visit. Similar to Kisanga, the majority comprised monkeys of the genera *Chlorocebus* and *Cercocebus*, but the meat of 934 black-and-white colobus (*Colobus angolensis*), 288 Thollon's red colobus (*Piliocolobus tholloni*) and 16 chimpanzees also was sold. While several species of primate, including chimpanzees and bonobos, are used occasionally for medicinal purposes in DRC, no quantitative data are available on how this affects wild populations (*Alves et al., 2010*). Alarmingly, the Red List assesment for Grauer’s gorilla reports an 80% decline in 20 years from almost 18,000 to fewer than 4,000 in 2015, caused principally by bushmeat hunting (*Plumptre et al., 2016*).

**Indonesia: live trade**

The trade in live primates in Indonesia occurs openly in dozens of wildlife markets, especially on the islands of Sumatra, Java and Bali. Primates are traded as pets in other parts of Indonesia as well, including Indonesian Borneo and on Sulawesi, but numbers are smaller (e.g. *Jones-Engel, et al., 2005*). *Shepherd* (*2010*) found 1,953 primates of 10 species for sale during 66 visits to bird markets in Medan, northern Sumatra. The most common species were long-tailed macaques (774 individuals), the greater slow loris (*Nycticebus coucang*, 714 individuals) and pig-tailed macaques (*Macaca nemestina,* 380 individuals). Some 1,300 primates of 8 species were recorded during 51 surveys in six markets on Java and Bali (*Nijman et al., 2017*). As in Sumatra, the most commonly traded species were long-tailed macaques (mainly infants and juveniles) and greater slow lorises (equal proportion of adults and young). Numerous primates, most notably gibbons and orangutans, have been confiscated by the authorities and taken to rescue centres (*Nijman, 2009*; *Nijman, Martinez & Shepherd, 2009*).

**Indonesia: medicinal and bushmeat trade**

In large parts of Indonesia, the majority of the local population is Muslim and individuals adhere to the principle that primates are unfit for human consumption. In parts of Indonesia where Muslims are a minority, primates are consumed, and they are traded as bushmeat. Large-scale commercial trade in primate meat (long-tailed and pig-tailed macaques, and Sulawesi crested macaques (*Macaca* *nigra*) have been reported from southern Sumatra and northern Sulawesi (*KSBK, 2002*; *Lee et al., 2005*). Based on the number of restaurants and specialized slaughterhouses in southern Sumatra, hundreds of macaques are killed monthly to meet the demand. Long-tailed macaques and, to a lesser degree, pig-tailed macaques are offered for sale in wild-meat restaurants in many of the larger cities in western Indonesia, especially in areas with a large Christian population (e.g. Medan, Pekanbaru, Jakarta). While trade in Sulawesi macaques in wild meat markets has been quantified, with typically between 0 and 5 carcasses available for sale during each visit (*Hilser et al., 2013; Lee et al., 2005*), data on turnover are lacking. Thus, it is difficult to determine the actual number involved. *Shekelle & Salim* (*2009*) reported that on Sunday afternoons, after Church, spit-roasted Sangihe Island tarsiers *Tarsius sangirensis*, were eaten communally as a snack. It is unclear to what extent this involved the commercial trade. in Indonesian Borneo, Bornean orangutans are killed for a variety of reasons but the main one appears to be for food (54% of the respondents that gave a reason indicated that this was their main motivation) and only very small proportions of respondents interviewed reported that killing occurred for traditional medicine or to sell infant orangutans (*Meijaard et al., 2012*). There was no indication that orangutan meat or medicine was traded commercially. Slow lorises are traded locally throughout Indonesia for medicinal purposes, (although numbers are dwarfed by the trade in live individuals; *Nekaris et al., 2010*). In general, the medicinal trade in primates is limited in Indonesia.

**Madagascar: live trade**

It appears that in recent years the number of lemurs kept as pets has increased throughout Madagascar. *Reuter* *et al*., (*2016*) reported ~30,000 pet lemurs of at least 16 species were identified over a three-year period. Most of these were the larger species including *Eulemur* spp., *Lemur catta* and *Varecia* spp. and excluded taxa that have taboos associated with them such as the aye-aye. The conditions in which these pet lemurs are being kept are suboptimal, resulting in short lifespans thus exacerbating the negative effect the pet trade has on wild populations (*Reuter & Schaefer, 2016*).

**Madagascar: medicinal and bushmeat trade**

Across parts of Madagascar, lemurs have been hunted for centuries, both for subsistence and trade (e.g. *Stiles, 1998*). Until relatively recently, however, the commercial trade in lemurs for meat was not considered to be a conservation concern. About a decade ago this changed, following political crisis and instability, and lemurs were traded as a premium meat (*Barrett & Ratsimbazafy, 2009*). While in the past many species of lemur were protected by taboos that prevented them from being killed, these taboos have eroded rapidly in recent years (*Jenkins et al., 2011*). The larger diurnal species such as the black-and-white ruffed lemur, Indri and sifaka are targeted, but the brown lemurs (*Eulemur* spp.) also are hunted (*Golden, 2009; Jenkins et al., 2011*; *Razafimanahaka et al., 2012*). While the emphasis of most studies has been on the larger species of lemur, even smaller species such as mouse lemurs (*Microcebus* spp*.*) are targeted. Individual hunters can capture up to 50 mouse lemurs a night, and therefore the impact on wild populations may be considerable (Gardner and Davies 2014). It appears that the commercial hunting and trade, especially in the larger species are unsustainable (*Golden, 2012*), but overall the threat that hunting and trade poses to the survival of lemurs needs to be more formally assessed (*Schwitzer et al., 2014*).

**Hunting**

The truth in DRC is that all primate species are hunted – pottos and galagos less often than the monkeys and great apes. This results in part from the fact that for most of the rural population, and for a large proportion of the urban population, other forms of animal protein are unavailable. DRC does not produce enough domestic meat to feed its population. And, although enormous quantities of Brazilian chicken and salted fish (saithe) from Norway are imported to the cities; rural people obtain their protein principally from wild animals (including freshwater fish and arthropods (*Bennett et al 2002*). Because wild meat farming tends to be far less efficient than raising domestic stock (in terms of growth rates and feed-conversion efficiency) (*Wilkie et al., 2016*; *Mockrin et al., 2005*) most workers have abandoned the idea of game ranching in forest environments and moving towards the farming of small domestic livestock such as guinea pigs and chickens (*Wilkie & Wieland, 2015*; *Wilkie et al., 2016*).

Hunting is a very serious problem and is exacerbated by increased access to forested areas using logging roads. However, in DRC hunters will actually *walk* over 100km to get to areas where they can find meat, as meat is very profitable compared to agricultural produce such as manioc tubers/ bananas which are hard to store (meat is smoked so it will last a very long time, especially if it gets re-smoked) and very inexpensive per kilo compared to meat.

In DRC, even twenty years ago, primates poached at a rate of 26 tons per year (the equivalent of about 5,000 individuals) in two districts in the Ituri forest, partially to meet the demand for the commercial bushmeat trade (*Nasi, Taber & Van Vliet, 2011*; *Wilkie et al., 1998*). On the Mentawai islands (West Sumatra), Indonesia, there are six endemic primates, all listed by the IUCN as threatened, and four of them are regularly hunted (the pig-tailed snub-nosed langur, *Simias concolor*; the Mentawai langur, *Presbytis siberu*; the Siberut macaque, *Macaca siberu*; and Kloss’ gibbon, *Hylobates klossii*), with an off-take estimated at 4,800 to 9,700 per year (*Quinten et al., 2014*).

**Human-modified landscapes and infectious diseases**

Two diseases that originated in Africa, Ebola virus disease (EVD) and yellow fever (YF), are among the most virulent human IDs that have impacted primate populations. In the case of YF, African primates are more resistant to the virus than their Neotropical counterparts, among which howler monkeys are the most sensitive (*Bugher, 1951*; *Vasconcelos, 2017*). Thousands of howler monkeys died during an epizootic outbreak in 2008 and 2009 in the state of Rio Grande do Sul (*Almeida et al., 2012*), leading to the reclassification of the conservation status of *A. caraya* from Vulnerable to Endangered. A subsequent ongoing YF epizootic event that began in 2016 in southeast Brazil caused the death of thousands of primates of many species (*Bicca-Marques et al., 2017*). These YF epizootics in Brazil also highlighted the importance of effective communication between scientists and the mass media to avoid misinformation that often results in local people, who afraid of contracting the disease, kill primates (*Bicca-Marques & Freitas, 2010*; *Bicca-Marques & Calegaro-Marques, 2014*; *Bicca-Marques et al. 2017*). In the case of YF, primates are neither a vector for human transmission, nor a reservoir of the virus. Harming and killing wildlife, including primates, are illegal activities in Brazil, but go underreported (*Bicca-Marques & Freitas, 2010*; *Bicca-Marques et al., 2017*).

EVD and YF also illustrate the most prevalent modes of transmission of pathogenic viruses and protists infecting primates (e.g. contact-borne and vector-borne, *Pedersen et al., 2005*), and the challenges of controlling epizootics and the spillover to humans. EVD infection occurs via contact with the blood or body fluids of infected individuals (*Gogarten et al., 2017*). Given the current lack of a vaccine and of any other effective strategy of controlling EVD in wildlife (*Nunn & Gillespie, 2016*), avoiding the consumption of bushmeat is necessary to prevent spillover to people (*Chapman, Gillespie & Goldberg,* *2005*). In the case of the mosquito-transmitted YF, there also is no management strategy to guard potential New World primate hosts. However, a focus on the infectious agent (the virus) via human vaccination for YF should be sufficient to reduce people’s concerns with the disease and the consequent risk of human-directed aggression against primates. Public health campaigns focused on vaccinating large numbers of humans can contribute to decreasing disease spread or its speed in fragmented landscapes, although the role of the vector-reservoir mosquitoes (*Consoli & Oliveira, 1994*), humans, and other (non-primate) wild hosts remains poorly understood. Therefore, developing effective vaccines against pathogenic agents and promoting immunization campaigns represent a critical human public health strategy that has direct positive implications for wildlife conservation (*Bicca-Marques et al., 2017*).

Unfortunately, our knowledge of the influence of infectious diseases on primate conservation is highly biased toward diseases that also are lethal to humans. Great apes are especially vulnerable to such diseases (*Gilardi et al., 2015*). Information also comes mainly from wealthier countries that possess the human capital and the scientific expertise to investigate the causes of wild primate deaths. In this regard, the existence of wildlife disease surveillance programs and the quality of a country’s public healthcare programs have direct implications for primate conservation. The scarcity of information hampers our ability to develop holistic conservation strategies for Brazil, Indonesia and Madagascar. In DRC, the size of the country and lack of infrastructure also hinder any nationwide conservation strategy. In DRC, the ability of primates to survive at ecotourism sites or near human habitation as urban commensals, where direct contact with humans and domesticated animals is higher and where supplementation and feeding on garbage may be common, increases exposure to diseases. These risks are higher for more terrestrial species (*Bicca-Marques & Calegaro-Marques, 2014*) and their conservation implications are particularly serious for those with low reproductive rates. Forest degradation and fragmentation have increased the level of parasitism in some species of Malagasy lemurs (*Microcebus murinus*; *Raharivololona & Ganzhorn, 2009*, *Eulemur flavifrons*; *Schwitzer et al., 2010*). This fact must become a focus of conservation studies and conservation policy.

**Human population**

**Population density distribution in each country**

In Brazil, areas of high population density are concentrated near the Atlantic coast, which has lost almost 88% of its original forest (*Ribeiro et al., 2009*). In Indonesia, the islands of Java and Sumatra are the most densely populated regions. In contrast, population density is more evenly distributed across DRC and Madagascar, with a single major concentration in the areas surrounding the capital cities of Kinshasa and Antananarivo (Available maps at. http://worldpopulationreview.com/countries/ (accessed 21 January 2018).

**Ethnic groups in each country**

Each of the four countries is characterized by a multiethnic component. Below we provide a summary of each multiethnic society taken from estimates for 2010 from the CIA Factbook (available at https://www.cia.gov/library/publications/the-world-factbook/ (accessed 20 January 2018).

| **Brazil**: white 47.7%, mulatto (mixed white and black) 43.1%, black 7.6%, Asian 1.1%, indigenous 0.4%. |
| --- |
| **DRC**: over 200 African ethnic groups of which the majority are Bantu; the four largest tribes - Mongo, Luba, Kongo (all Bantu), and the Mangbetu-Azande (Hamitic) make up about 45% of the population. |
| **Madagascar**: Malayo-Indonesian (Merina and related Betsileo), Cotiers (mixed African, Malayo-Indonesian, and Arab ancestry - Betsimisaraka, Tsimihety, Antaisaka, Sakalava), French, Indian, Creole, Comoran. |
| **Indonesia**: Javanese 40.1%, Sundanese 15.5%, Malay 3.7%, Batak 3.6%, Madurese 3%, Betawi 2.9%, Minangkabau 2.7%, Buginese 2.7%, Bantenese 2%, Banjarese 1.7%, Balinese 1.7%, Acehnese 1.4%, Dayak 1.4%, Sasak 1.3%, Chinese 1.2%, other 15%. |

**Corruption, governance quality and primate conservation**

**DRC**

DRC has a patronage system where the profits of “unofficial economic activities” flow upwards to the top of the chain of command (*Baaz & Olssen, 2011*) and the chaos within different branches of this system serve to maintain the status quo that only benefits the wealthy few (*Nlandu Mayamba, 2012*). Negotiation is key to all interactions between State officials and the ordinary citizen (*Trefon, 2009, 2010, 2013*) and therefore environmental issues are not treated in the same way as in many other countries (Trefon, 2016).

**Brazil**

Recent political events in Brazil highlight the extreme level of government corruption in that country. Companies and people who committed crimes and environmental infractions in Brazil have donated almost $20 million USD to more than half of the House of Representatives in the last election, among those having receiving bribes is the current Minister of the Environment. See reports below documenting this situation.

http://www.nature.com/news/political-upheaval-threatens-brazil-s-environmental-protections-1.20955

http://reporterbrasil.org.br/2018/01/maioria-dos-deputados-recebeu-doacao-de-desmatadores-como-isso-reflete-na-sua-atuacao/

https://news.mongabay.com/2017/09/brazil-a-world-champion-in-political-and-environmental-devastation-commentary/

https://www.theguardian.com/commentisfree/2017/may/23/the-guardian-view-on-brazilian-corruption-the-public-deserve-a-voice

**Protected areas**

**Brazil**

In 1976, there were only two national parks in the Brazilian Amazon, but in that year the government produced a scientifically-based proposal for an Amazon-wide protected area network based on the biogeography of the region (phytogeographic regions and areas of endemism) (Brazil, MA-IBDF & FBCN, 1979; *Mittermeier et al., 2005*; *Rylands & Brandon, 2005*; *Wetterberg, Prance &* Lovejoy, 1981; *Wetterberg et al., 1976*). This was followed in 1990 by a conservation priority-setting workshop for the entire Amazon organized by the Brazilian Institute for the Environment (Ibama), the Brazilian National Institute for Amazon Research (INPA), and several international and national conservation NGOs (*Prance, 1990*; *Rylands, 1990*; *Rylands, Huber & Brown Jr., 1991*). Brazil’s Ministry of the Environment subsequently held priority-setting workshops for all of the country’s major biomes (1998-2000), including the primate-rich Atlantic Forest and the Brazilian Amazon, which determined the location of 900 priority areas for biodiversity conservation (*Brazil, MMA. 2002*; *Mittermeier et al., 2005*; *Rylands & Brandon, 2005*). In 2000, Brazil established a formal, unified system for federal, state and municipal parks and reserves (the National System for Protected Areas – SNUC). Parks and reserves were used as the keystone—contiguous protected areas of differing categories for the establishment of biodiversity corridors—(*Anonymous, 2003; Ayres et al., 2005*). Of note for the Amazon is the program for the creation of protected areas in the 23 Amazonian eco-regions identified by and supported by the World Wildlife Fund – Brazil and The World Bank—the Amazon Protected Areas (ARPA) program of the Brazilian Ministry of the Environment (*Ferreira et al., 2001*). The SNUC aimed to increase the area of the Amazon rainforest under federal protection to 500,000 km². In 2005 it was estimated that there were 478 Brazilian federal and state strictly protected areas, totaling 37,019,697 ha, and 436 sustainable-use protected areas, totaling 74,592,691 ha (*Rylands & Brandon, 2005*).

**DRC**

DRC has 11% of its land within 90 protected areas and plans to increase the coverage to 17% (following Aichi Target 11: *CBD, 2011*). Five of the PAs are World Heritage Sites, but all are classified as In Danger. Some are extremely large: the Salonga National Park is over 36,000 km^2^. All are key for wildlife conservation. However, several primate species are either outside PAs or most of their population is found only within PAs (both gorilla subspecies in DRC fall into this category). Those PAs that are the recipients of international funding are reasonably well-staffed and resourced, but if a PA has no partner organization, it tends to be rapidly hunted out or in the savannahs of the south, converted to smallholder agriculture.

Starting in 2000, twelve very large conservation landscapes were created throughout Central Africa. Each contained one or more protected areas at their core, surrounded by community lands and logging concessions. This has proven to be the most successful model for wildlife conservation in the region in general, including for primate conservation (*Yanggen et al 2010, USAID 2012*). These landscapes cover 685,400 km^2^ (almost 40% of the Congo Basin) and include over 30 protected areas (*CBFP, 2006*); because of its large size, DRC has six of the twelve landscapes. This approach was instigated by USAID’s CARPE program and is now in its third phase, as part of the wide-reaching CBFP (Congo Basin Forest Partnership that brings many organizations and all the range states together under one umbrella). The inclusion of this landscape approach is now being applied to DRC’s carbon reduction program (*Fobissie, 2015*), and should benefit primates by maintaining old-growth forests.

**Community managed forests, habitat restoration and landscape connectivity**

Considering the widespread transformation of primate habitats to anthropogenic vegetation, there is a need for more work to be done on landscape approaches to primate conservation that include a mosaic of land-use practices where more flexible primate species (e.g. chimpanzees but not bonobos and gorillas; *Hockings et al., 2015*) can survive if they are not hunted (this would not be possible in DRC as all wildlife is hunted for meat and there is almost no domestic animal protein alternative widely available). Primate behavior does not always conflict with human interests, and interdisciplinary research has the potential to greatly improve our understanding of the complexities of human-primate interactions in shared landscapes (*McLennan et al. 2017; Mukul & Saha, 2017*; *Newmark et al., 2017*) (see Text S1 for specific examples of primates in agroecosystems in the four countries).

A global review showed that 57 primate taxa from Mesoamerica, South America, Sub-Saharan Africa (including Madagascar), and Southeast Asia, used 38 types of agroecosystems as temporary or permanent habitats and that about 40% of the primates recorded in these agroecosystems were classiﬁed as threatened (*Estrada et al., 2012*). At present, it remains unclear how long primate populations can survive and reproduce successfully in agroecosystems and therefore caution must be used before advocating this as a sustainable solution to primate population decline. Notwithstanding, in Brazil’s southern Atlantic Forest, groups of the black and gold howler monkey are present in eucalyptus plantations and black-fronted titi monkeys (*Callicebus nigrifrons*) are reported to inhabit eucalyptus in Canareira State Park (Sao Paulo, Brazil). In both cases, the primates use eucalyptus trees as sources of food (*Trevelin et al., 2007*). Shaded-cacao agroforestry, known in Brazil as cabruca, is the predominant habitat type throughout the eastern portion of the distribution of the endemic and endangered golden-headed lion tamarin (*Raboy et al., 2010*).

In eastern Madagascar, seven sympatric species of Strepsirhini (Gmelin’s woolly lemur, *Avahi laniger*; Geoffroyi’s dwarf lemur, *Cheirogaleus major*; Eastern lesser bamboo lemur, *Hapalemur griseus*; Indri, *Indri indri*; common brown lemur, *Eulemur fulvus*; weasel sportive lemur, *Lepilemur mustelinus*; rufous mouse lemur, *Microcebus rufus*) are reported to live in eucalyptus plantations. Some of these species use the plantations mainly for resting and to travel from one patch of native forest to another, but others feed on leaves, fruits, and ﬂowers in these agroforests, including the ﬂowers of *Eucalyptus sp*. (Ganzhorn, 1987). The same study reports groups of Verreaux’s sifaka (*Propithecus verreauxi*) as permanent residents of mixed mango (*Mangifera indica*) and vavaloza trees (*Stereospermum arcuatum*) where they feed on the fruit and leaves of mangos and on the ﬂowers and leaves of *S. arcuatum*. Other primates present in these mixed plantations include *A. laniger*, *L. mustelinus*, and *E. fulvus*. (*Ganzhorn, 1987; Ganzhorn & Abrahams, 1991*). Black lemurs (*Eulemur macaco macaco*) were reported living in mixed plantations of mango, coffee, coconut, and papaya in northwestern Madagascar. They feed on mangos, papayas, palm fruits, and ﬂowers of the Dypsis palm (*Dypsis* spp.), and cross berry fruits (*Grewia* spp.) (*Simmen et al., 2007*). Sheth’s dwarf lemurs were found to live in vanilla plantations in northern Madagascar (*Hending et al., 2017*).

In Indonesia, Bornean orangutans can survive, at least temporarily, in logged forests, *Acacia* plantations, and oil palm plantations (*Meijaard et al., 2012*). In natural rubber and oil palm plantations in Sumatra, seeds, leaves, flowers, and bark are used as a food source by orangutans (*Pongo abelii*) and purple-faced leaf monkeys (*Trachypithecus vetulus*) (*Ancrenaz et. al. 2015*; *Harich & Treydte, 2016*). In Gulung Palung National Park, Kalimantan, maroon leaf monkeys (*Presbytis rubicunda*) and agile gibbons (*Hylobates agilis*) are found in agroforests (*Salafsky, 1993*). Maroon leaf monkeys, southern pig-tailed macaques *(Macaca nemestrina)*, and siamangs (*Symphalangus syndactylus*) are present in rubber (*Hevea brasiliensis*) and dammar (*Shorea javanica*) agroforests, and in durian (*Durio zibethinus*) agroforests in Sumatra where they occur in similar densities as they do in primary forests (*Michon & de Foresta, 1995*). Dian’s tarsiers (*Tarsius dentatus*) in Sulawesi, Indonesia, occupy mixed-species plantations of cacao and gliricidia (*Gliricidia sepium*) with interspersed patches of dense shrub, bamboo (*Bambusa* spp.), Cogon grass (*Imperata cylindrical*), and corn outside of native forests (*Merker, Yustian & Mühlenberg, 2005*). It appears that limited human disturbance does not pose a major threat to Dian’s tarsiers. In Batang Serangan northern Sumatra, a small population of Sumatran orangutans are reported living for several decades in a mixed agroforest system composed of oil palm (*Elaeis guineensis*), rubber trees (*Hevea brasiliensis*), and remnant forest. They feed on jackfruit and durian, among others introduced food items (*Campbell-Smith et al., 2010*). In addition to orangutans, other primates living in this agroecosystem are Thomas’s langur (*Presbytis thomasi*), common long-tailed macaques (*Macaca fascicularis fascicularis*), southern pigtailed macaques (*Macaca nemestrina*), Lar gibbons (*Hylobates lar*), and Grifﬁth’s silver langurs (*Trachypithecus villosus*) (*Campbell-Smith et al., 2010*). Overall, these data suggest that some primates can exploit certain types of agroecosystems when faced with anthropogenic habitat loss, fragmentation, and degradation. However, this may not be a viable long-term solution in the face of continued loss of forest habitat (*Estrada et al., 2012*).

**Primate rewilding**

In addition to the IUCN guidelines for reintroduction of wildlife (*IUCN/SSG, 2013).* The IUCN has published *Guidelines for Nonhuman Primate Re-introductions* in 2002, *Best Practice Guidelines for the Re–introduction of Great Apes* in 2007 (in English, French and Bahasa Indonesia), and *Gibbon Rehabilitation, Reintroduction and Translocation Guideline*s in 2015. All can be downloaded from the IUCN Library Portal.

**Socially‒oriented conservation actions for averting local extinction threats to primates**

**DRC**

In DRC, a number of international conservation NGOs are working in and around all of these conservation landscapes, with coordination offices in the capital. Some of these NGOs have been working in DRC for over 30 years. Many of the small, locally-based environmental NGOs across DRC, are usually focused on a particular reserve and the surrounding community lands, or even on one village or town. Their activities are small-scale and tend towards conservation education /advocacy, and small agricultural and microfinance development activities, although in general they collaborate with international NGOs, who are able to leverage funding for such partnerships. In DRC, the large NGOs organize regular wildlife surveys in DRC’s protected areas and their buffer zones, collecting data on large mammals and human impact. As research assistants, Congolese University graduates find their feet as field officers, researchers, and project and program managers. Sustainable-livelihood programs, land-use planning, and improved governance are integral components of these conservation landscape projects.

In DRC, the Centre de Rehabilitation ​des Primates ​​de Luiro was created in 2002 by two Congolese Institutions; Institut Congolais pour la Conservation de la Nature (ICCN) and the Centre de Recherché en Sciences Naturelles (CRSN). The Centre for the Rehabilitation for Primates is an important conservation tool for Primates in DRC. Currently (2017) they care for 72 chimpanzees and 92 monkeys of 11 different species, all of them victims of the pet trade and poaching. Without centers like this, no confiscation would be possible, therefore the CRPL is a key factor for law enforcement.  The education and sensitization programs of CRPL reach more than 3.000 people per year. CRPL is an accredited sanctuary of the [Pan Africans Sanctuary Alliance](https://www.pasaprimates.org/)(PASA) and [Global Federation of Animals Sanctuaries](http://www.sanctuaryfederation.org/gfas/)(GFAS) (see https://www.lwiroprimates.org/about for details)..

**Brazil**

Primate conservation in Brazil has benefitted from the International Committees for Conservation and Management (ICCM) – forums of institutions and specialists involved in the conservation of threatened species –, first, in the early 1980s, as informal groups, and later from the 1990s as official organizations to advise the Brazilian Government. This involvement has been crucial for threatened and charismatic species of the Atlantic Forest, such as the lion tamarins and the muriquis. The pioneer ICCMs were those for the lion tamarins, which promoted strategic planning for the conservation of the four *Leontopithecus* species and stimulated the creation of national NGOs commited to their conservation (*Holst et al. 2006*; *Rambaldi et al. 2008, 2012*; *Kierulff et al.2012*). The NGO Associação Mico-Leão-Dourado (AMLD, Golden Lion Tamarin Association) was created in 1992 specifically for promoting the conservation of the golden lion tamarin (*Leontopithecus rosalia*), including habitat protection, metapopulation management, forest restoration, conservation education, sustainable ecotourism, and scientific research (*Oliveira, Grativol & Ruiz-Miranda, 2008*; *Kierulff et al., 2012*). The metapopulation management program – including reintroductions, supplementations, and translocations – of golden lion tamarins, for instance, is probably the world’s most successful primate project of this kind, and currently the wild population has increased to *ca* 3,200 individuals from an estimated 100 to 200 in 1975 (*Coimbra-Filho & Mittermeier, 1976*; *Kierulff et al., 2012*). This program functioned with the support of the Centro de Primatologia do Rio de Janeiro (CPRJ) was was created in 1979 by Adelmar Coimbra-Filho. This was conceived as a governamental center to manage golden lion tamarins in captivity focusing on their reproduction and reintroduction (*Coimbra-Filho, 2004*; *INEA, 2015*). This species became nationally known in 2002, when it was first depicted on the 20 reals (the Brazilian currency) bill. Two congenerics of golden lion tamarins, the black (*L. chrysopygus*) and the black-faced (*L. caissara*) lion tamarins have been the focus of the Instituto de Pesquisas Ecológicas (IPÊ, Institute for Ecological Research), also founded in 1992. The leading role played by IPÊ in research, management and conservation projects for these species also has played a critical role in their conservation (*Rambaldi et al., 2008*; *Rezende, 2014*).

The creation, in 1994, of the Instituto de Estudos Socioambientais do Sul da Bahia (IESB), benefited research and conservation for the golden-headed lion tamarin (*L. chrysopygus*) (*Rambaldi et al., 2008*) and the yellow-breasted capuchin monkey (*Sapajus xanthosternos*) (*Kierulff et al., 2005*) in the Atlantic forest of Southern Bahia State. More recently, the Instituto Pri-Matas para a Conservação da Biodiversidade (Pri-Matas, Pri-Mates Institute for Biodiversity Conservation) has invested several years of work in removing an invasive population of the golden-headed lion tamarin (*L. chrysomelas*) from forests near (*ca* 50 km away from) the distribution of the golden lion tamarin in the State of Rio de Janeiro. This has served to eliminate a potential source of hybridization, resource competition, and disease spillover that would compromise the conservation of golden lion tamarins (*Kierulff, 2010*). The Bicho do Mato Instituto de Pesquisa (Bicho do Mato Research Institute) in collaboration with the Antwep Zoo has, since 2011, led projects on ecology and behavior of the golden-headed lion tamarin (*L. chrysomelas*) in different habitats in Bahia, Brazil. The initiatives for conservation of the muriquis (*Brachyteles* spp.) followed some of the well successful efforts targeting the lion tamarins, such as a Population and Habitat Viability Analysis (PHVA) and the creation of a ICCM (*Rylands et al., 1998; Talebi et al., 2011*). Also, some NGOs were founded specifically to promote research, education and conservation projects for the muriqui. The Pro-Muriqui Institute ([promuriqui.org.br](http://promuriqui.org.br/)), founded in 2000 by recommendation of the Muriqui PHVA 1998 and led by the Conservation Breeding Specialist Group, acts to coordinate and execute southern muriqui research within the large extensions of forest still existing in São Paulo State (*Talebi et al., 2011*). The Preserve Muriqui institute ([http://www.preservemuriqui.org.br](http://www.preservemuriqui.org.br/)) leads the conservation efforts of the Caratinga Biological Station-Private Reserve Feliciano Miguel Abdala in the state of Minas Gerais (*Strier, 2007*).  While the Muriqui Instituto de Biodiversidade ([https://www.facebook.com/muriquibiodiveridade/](https://www.facebook.com/muriquibiodiveridade/)) has, since 2015, led projects mainly in the state of Minas Gerais.

In the Brazilian Amazon, uacaris (*Cacajao calvus*) have received the special attention of the Mamirauá Institute since the pioneer work of Ayres (1986) who also contributed to conservation efforts targeting the pied tamarin (*Saguinus bicolor*) (*Ayres, Mittermeier & Constable, 1982*). The latter species has been the focus of the ICCM since the late 1990s.  The strategies for the conservation of threatened primates in Brazil are currently organized as part of National Action Plans (NAPs), coordinated by the Centro Nacional de Pesquisa e Conservação de Primatas Brasileiros (CPB, National Center for Research and Conservation of Brazilian Primates) of the Instituto Chico Mendes de Conservação da Biodiversidade (ICMBio, Chico Mendes Institute for Biodiversity Conservation), which is part of the Brazilian Environment Ministry, and implemented by several institutions including NGOs, universities and other governamental and private organizations (*Jerusalinsky, 2016*). Examples of these NAPs are those for the muriquis (*Jerusalinsky et al., 2011*), the pied tamarins, and the Atlantic forest mammals (*Escarlate-Tavares, Valença-Montenegro & Jerusalinsky, 2016*), which include 13 primate species.

From the 1960s, some initiatives – such as CITES and resolutions from the *World Association of Zoos and Aquariums* (WAZA) – were decisive in reducing the illegal trade of threatened primates such as the lion-tamarins (*Leontopithecus* spp.), which were previously highly commercialized mainly in zoos or private colections (*Ballou et al., 2002*).

**Indonesia**

In Indonesia, in 2005 a group of local conservationists initiated a project in Central Java to conserve the Javan surili (*Presbytis comata*) (*Setiawan et al., 2010*) ,and Javan gibbons (*Hylobates moloch*) (*Setiawan et al., 2012*). In 2012 an official protection team, Swaraowa was founded (*http://swaraowa.com/about-us/*). Swaraowa works with local communities to promote sustainable conservation of Javan gibbon through the shade grown coffee project. These gibbons are distributed outside of conservation area and face high levels of human activities and anthropogenic disturbance. The Coffee and Primate Conservation Project is a grassroots initiative to develop a sustainable economy for conservation, namely shade grown coffee, as one of the non-timber forest products in this area. This initiative has the potential to result in a sustainable ecological and economic model to protect endangered primates and their habitat, as well as provide a source of income for the local community.

Also, in Indonesia, in 2011 the Little Fireface Project (*http://www.nocturama.org/en/welcome-little-fireface-project/*) was established to examine the multi-faceted problems facing wild slow lorises (*Nekaris, 2016*). A long-term field project was started in Garut, West Java, where the Critically Endangered Javan slow loris (*Nycticebus javanicus*) is endemic. Little Fireface is the local Sundanese name for slow lorises. Java wide surveys have been conducted, and weekly conservation education sessions are held and assessed (*Nekaris et al., 2018*). Conservation is targeted to a wide range of audiences and stakeholders, with annual training sessions for law enforcement officers and coordinated biannual events in villages close to wild slow loris populations. These events are designed to increase pride in this endemic species (*Nekaris, 2016*).

**Madagascar**

In Madagascar, two university ‘departments’ focus their training programs on primatology, namely the "Anthropologie Biologique et Evolution” of the University of Antananarivo and the "Ecologie des Primates" of the University of Mahajanga. Their main objectives are to provide students with in-depth knowledge of the natural history of primates, especially lemurs, so that they can understand the biological and economic importance of this taxon for the country, undertake research endeavors, and promote conservation efforts.

(See: https://www.univ-antananarivo.mg/IMG/pdf/lesystemelmd_universitedetana.pdf

lemurconservationnetwork.org/how-to-help/support-research/

https://www.univ-mahajanga.edu.mg/fste/masters/)

**Invasive species**

Primate species released outside of their natural range threaten other species including native primates. The problem is localized to some regions but has increased in the last several years, sometimes with serious consequences to the native primates. Introduced primates often compete for resources with native species, hybridize, or result in the local extirpation of native species. In Rio de Janeiro, Brazil, introduced marmosets (White-tufted-ear or common marmoset *Callithrix jacchus* and Black-tufted-ear marmoset *Callitrix penicillata*) compete for food with golden-lion tamarin, *Leontopithecus rosalia* (*Oliveira, & Grelle 2012; Ruiz-Miranda et al., 2006*). Hybrids between the two invader marmosets and the native buffy-tufted marmosets (*Callitrix aurita*) have been recorded in the wild in Rio de Janeiro and São Paulo States (*Detogne et al., 2017; Carvalho et al., 2013; Melo et al., 2015; Nogueira et al.,2011; Port-Carvalho & Kierulff, 2009*). *C. aurita* is classified as Vulnerable by IUCN/SSC Red List and the Brazilian Official List of Threatened Species, and in some protected areas part of some of wild population are considered to be hybrids (*Brasil, 2014; Detogne et al., 2017; Carvalho et al., 2013; The IUCN Red List of Threatened Species, 2017; Nogueira et al., 2011*). It was estimated that the range of *C. aurita* is likely to be reduced by at least 50% within the next 18 years due to habitat loss and introduction of invasive marmosets (*Detogne et al., 2017; Norris et al., 2011; Pereira et al., 2008*). Another example in Rio de Janeiro is the introduction and invasion of exotic golden-headed lion tamarins *Leontopithecus chrysomelas* (Golden-headed lion tamarin) within the natural range of *Leontopithecus rosalia* (Golden lion tamarin) (*Kierulff, 2010*). Both species are classified as “Endangered” on the IUCN/SSC Red List and the Brazilian Official List of Threatened Species (Brasil, 2014; The IUCN Red List of Threatened Species, 2018). Currently, the two species are separated in different forest fragments, but these fragments are not far from each other.. The chances are high that the two species will hybridize, as has been documented in captivity (*Coimbra-Filho & Mittermeier 1976*). There also is a risk that the golden-headed lion tamarins will introduce diseases previously absent from the golden lion tamarin population. Invader species also impact biodiversity negatively by consuming the eggs and chicks of endangered forest birds and may compete with native birds for resources. In Indonesia, crab-eating macaques (*Macaca fascicularis*) introduced into Tinjil Island and Papua*,* feed on sugar and other crops, negatively affecting agriculture and livelihoods local farmers (*Global Invasive Species Database, 2018*). These monkeys can be aggressive to humans and these areas of primate-human conflict generally result in the extermination of the primate population.

**Primate societies**

Because of the recent growth in trained primatologists in the four countries, their conservation concerns have recently led to the creation of professional societies that can more effectively articulate conservation concerns with local governments, NGOs and communities. Some of these scientific associations have partnered with continent-wide societies such as the African Primatological Society https://www.facebook.com/.African.Primatological.Society, the Latinoamerican Society of Primatology (http://www.slaprim.org/) and globally with the International Primatological Society (http://www.internationalprimatologicalsociety.org/affiliatedsocieties.cfm). These partnerships are likely to result in regional and global action in favor of primate conservation (see

https://www.conservationevidence.com/data/index)

Below we list such societies.

**Brazil:** The Brazilian Society of Primatology (http://www.primatologia.org.br/) is a well-established society.

**Indonesia:** The main Indonesian society doing primate conservation is ProFauna (http://www.profauna.net/en/about-profauna/what-is-profauna#.WnHkWWnwaM8) which used to be  Konservasi Satwa Bagi Kehidupan (founded in 1934 in Java).

**Madagascar:** The Malagasy Primatological Society (GERP (Groupe d’étude et de recherche sur les primates de Madagascar; http://lemurconservationnetwork.org/organization/gerp/:

http://gerp.squarespace.com. In general, this is a great resource for (most) organizations involved in lemur conservation: http://lemurconservationnetwork.org/about/

In 1994, ten Malagasy primatologists dedicated to protecting wildlife established a research institution in the form of an association called Groupe d'Etude et de Recherche sur les Primates de Madagascar (GERP). This organization has grown considerably and is now comprised of researchers, teachers, students, founding members, donors, and consultants. Today, GERP has 111 members including 15 foreigners. The main goal of GERP is to share knowledge and skills in order to preserve biodiversity for future generations (http://gerp.squarespace.com/who-we-are).

**DRC:** no society exists.

**Other regional associations**

Southeast Asian Primatological Association: http://seapa.atspace.com/

Section on Great Apes: http://www.primate-sg.org/section_great_apes/

Section on Small Apes: http://www.gibbons.asia/

The Silvery Gibbon Project: https://silvery.org.au/

Orangutan Foundation Intl: https://orangutan.org/

**REFERENCES**

**ABIEC BRAZIL.** **2016**. Brazilian Livestock Profile Annual Report 2016; http://www.newsprime.com.br/img/upload2/2016_FolderPerfil_EN.pdf

**Ahrends A., Hollingsworth PM, Ziegler AD, Fox JM, Chen H, Su Y, Xu J. 2015**. Current trends of rubber plantation expansion may threaten biodiversity and livelihoods. *Global Environmental Change* **34:**48–58.

**Almeida MAB, Santos E, Cardoso JC, Fonseca DF, Noll CA, Silveira VR, Maeda AY, Souza RP, Kanamura C & Brasil RA.** **2012**. Yellow fever outbreak affecting *Alouatta* populations in Southern Brazil (Rio Grande do Sul State), 2008-2009. *American Journal of Primatology* **74**:68-76.

**Alves RRN, Souto WMS, Barboza RRD.** **2010**. Primates in traditional folk medicine: A world overview. *Mammalian Review* **40**:155–180.

**Ancrenaz M, Oram F, Ambu L, Lackman I, Ahmad E, Elahan H, Kler H, Abram NK, Meijaard E. 2015**. Of *Pongo*, palms and perceptions: a multidisciplinary assessment of Bornean orang-utans *Pongo pygmaeus* in an oil palm context. *Oryx* **49**:465-472.

**André C, Kamate C, Mbonzo P, Morel D, Hare B.** **2008**. The conservation value of Lola Ya Bonobo Sanctuary. In: Takeshi F, Thompson J, eds. *The bonobos: Behaviour, ecology and conservation*. Springer Press, 303-322.

**Anonymous**. **2003**. Amapá biodiversity corridor. *Neotropical Primates* **11**:191–192.

**Ayres JM.** **1986**. The conservation status of the white uakari. *Primate Conservation* **7**:22-26.

**Ayres JM, Mittermeier RA & Constable ID.** **1982**. Brazilian tamarins on the way to extinction? *Oryx* **16**:329-333.

**Ayres JM, Fonseca, GAB da, Rylands AB, Queiroz HL. Pinto LP de S, Masterson D, Cavalcanti RB.** **2005**. *Os Corredores Ecológicos das Florestas Tropicais do Brasil*. Sociedade Civil Mamirauá. 256p.

**Baaz ME, Olsson O. 2011**. Feeding the Horse: Unofficial economic activities within the police force in the Democratic Republic of the Congo. *African Security* **4**:223–241. DOI: 10.1080/19392206.2011.628629.

**Ballou JD, Kleiman DG, Mallinson JJC, Rylands AB, Valladares-Padua CB, Leus K. 2002.** History, management and conservation role of the captive lion tamarin populations. In: Kleiman DG, Rylands AB, eds. *Lion Tamarins: Biology and Conservation.* Smithsonian Institution Press, 95–114.

**Barrett MA, Ratsimbazafy J.** **2009**. Luxury bushmeat trade threatens lemur conservation. *Nature* **461**:470-470.

**Bennett EL, Eves HE, Robinson JG, Wilkie DS. 2002.** Why is eating bushmeat a biodiversity crisis? *Conservation in Practice* **3:**28-29.

**Bicca-Marques JC, Freitas DS.** **2010**. The role of monkeys, mosquitoes, and humans in the occurrence of a yellow fever outbreak in a fragmented landscape in south Brazil: protecting howler monkeys is a matter of public health. *Tropical Conservation Science* **3**:31-42.

**Bicca-Marques JC, Calegaro-Marques C.** **2014**. Parasite sharing between humans and nonhuman primates and the hidden dangers to primate conservation. *Zoologia* **31**:313-315.

**Bicca-Marques JC, Calegaro-Marques C, Rylands AB, Strier KB, Mittermeier RA, Almeida MAB, Castro PHG, Chaves ÓM, Ferraz LP, Fortes VB, Hirano ZMB, Jerusalinsky L, Kowalewski M, Martins WP, Melo FR, Mendes SL, Neves LG, Passos FC, Port-Carvalho M, Ribeiro S, Romano APM, Ruiz-Miranda CR, Santos EO, Souza Jr JC & Teixeira DS. 2017.** Yellow fever threatens Atlantic Forest primates. *Science Advances* 3:e1600946/tab-e-letters.

**Brasil, 2014.** Ministério do Meio Ambiente. Portaria n° 444, 17 de dezembro de 2014. 2014. Lista Nacional Oficial de Espécies da Fauna Ameaçada de Extinção. Diário *Oficial da República Federativa do Brasil. Brasília, DF, 17 de dez. de 2014. Seção I,* p.121–126.

**Brazil, MA-IBDF, FBCN.** **1979**. *Plano do Sistema de Unidades de Conservação do Brasil.* Ministério da Agricultura (MA), Instituto Brasileiro de Desenvolvimento Florestal (IBDF), and Fundação Brasileira para a Conservação da Natureza (FBCN), Brasília, Brazil.

**Brazil, MMA.** **2002**. *Biodiversidade Brasileira: Avaliação e Identificação de Áreas e Ações Prioritárias para Conservação, Utilização Sustentável e Repartição de Benefícios da Biodiversidade Brasileira*. Secretaria de Biodiversidade e Florestas (SBF), Ministério do Meio Ambiente (MMA), Brasília, Brazil.

**Bugher JC. 1951**. The mammalian host in yellow fever. In: Yellow fever. In: Strode GK, ed. McGraw Hill, 299-384.

**Campbell-Smith GA, Hubert VP, Simanjorang, Williams L, Linkie NM.** **2010**. Local attitudes and perceptions toward crop-raiding by orangutans (*Pongo abelii*) and other nonhuman primates in northern Sumatra, Indonesia. *American Journal of Primatology* **71**:1–11.

**Carvalho RS, Silva DA, Loiola, S, Pereira DG, Carvalho EF, Bergallo HG. 2013.** Molecular identification of a Buffy-tufted-ear marmoset (*Callithrix aurita*) incorporated in a group of invasive marmosets in the Serra dos Orgãos National Park, Rio de Janeiro— Brazil. *Forensic Science International: Genetics Supplement Series* **4**:230–231.

**CBD. 2011.** *Strategic Plan for Biodiversity 2011-2020: Aichi Targets*. CBD-UNEP. https://www.cbd.int/sp/

**CBFP. 2006.** Congo Basin Forest Partnership-The forests of the Congo Basin: State of the Forest 2006. Kinshasa, DRC: CBFP.

**Chapman CA, Gillespie TR & Goldberg TL.** **2005**. Primates and the ecology of their infectious diseases: how will anthropogenic change affect host-parasite interactions? *Evolutionary Anthropology* **14**:134-144.

**CIA 2017. https://www.cia.gov/library/publications/resources/the-world-factbook/geos/id.html**

**Coimbra-Filho AF. 2004**. Os primórdios da primatologia no Brasil. In: Mendes SL, Chiarello AG, eds. *A Primatologia no Brasil* – volume 8. Sociedade Brasileira de Primatologia. Vitória, Brasil. 11-35.

**Coimbra-Filho AF, Mittermeier RA. 1976**. Hybridization in the genus *Leontopithecus*, *Leontopithecus r. rosalia* (Linneus, 1766) x *L. r. chrysomaleas* (Kuhl, 1830) (Callitrichidae, Primates). *Revista Brasileira de Biologia* **36**:129-137.

**Consoli RAGB, Oliveira RL.** **1994**. Principais mosquitos de importância sanitária no Brasil. Fiocruz, Rio de Janeiro. 228 p. ISBN 85-85676-03-5.

**Da Silva FA, Canale GR, Kierulff MCM, Duarte GT, Paglia AP Bernardo CS. 2016**. Hunting, pet trade, and forest size effects on population viability of a critically endangered Neotropical primate, *Sapajus xanthosternos* (Wied‐Neuwied, 1826). *American Journal of Primatology* **78**:950-960.

**Debroux L, Hart T, Kaimowitz D, Karsenty A, Topa G, eds. 2007***. Forests in Post-Conflict Democratic Republic of Congo: Analysis of a Priority Agenda*. A joint report by teams of the World Bank, Center for International Forestry Research (CIFOR), Centre International de Recherche Agronomique pour le Développement (CIRAD), African Wildlife Foundation, Conseil National des ONG de Développement du Congo, Conservation International, Groupe de Travail Forêts, Ligue Nationale des Pygmées du Congo, Netherlands Development Organisation, Réseau des Partenaires pour l’Environnement au Congo, Wildlife Conservation Society, Woods Hole Research Center, World Agroforestry Centre and World Wide Fund for Nature. CIFOR, World Bank and CIRAD, Washington, DC. Available at https://www.cifor.org/publications/pdf_files/Books/BCIFOR0701.pdf

**Detogne N, Ferreguetti AC, Mello JHF, Santana MC, Dias AC, Mota NCJ, Gonçalves AEC, Souza CPS, Bergallo HG. 2017**. Spatial distribution of buffy-tufted-ear (*Callithrix aurita*) and invasive marmosets (*Callithrix* spp.) in a tropical rainforest reserve in southeastern Brazil. *American Journal of Primatology*. Available at <https://doi.org/10.1002/ajp.22718>.

**Dixon J, Gulliver A, Gibbon D. 2001.** *Farming Systems and Poverty: improving farmers' livelihoods in a changing world*. FAO and World Bank, Rome and Washington D.C. Avilable at [www.fao.org/3/a-ac349e.pdf](http://www.fao.org/3/a-ac349e.pdf)

**Dobrovolski R, Rattis L. 2014**. Brazil should help developing nations to foster agriculture and environmental protection. *Frontiers in Ecology and the Environment*, **12**:376-376.

**Do Nascimento RA, Schiavetti A, Montaño RAM.** **2013**. An assessment of illegal capuchin monkey trade in Bahia State, Brazil. *Neotropical Biology and Conservation* **8**:79-87.

**Dupain J, Nackoney J, Vargas JM, Johnson PJ, Farfán MA, Bofaso M, Fa JE.** **2012**. Bushmeat characteristics vary with catchment conditions in a Congo market. *Biological Conservation*, **146**:32-40.

**Ernst C, Mayaux P, Verhegghen A, Bodart C, Christophe M, Defourny P.** **2013.** National forest cover change in Congo Basin: deforestation, reforestation, degradation and regeneration for the years 1990, 2000 and 2005**.** *Global Change Biology* **19**:1173-1187. DOI: 10.1111/gcb.12092.

**Escarlate-Tavares F, Valença-Montenegro MM, Jerusalinsky L. (orgs.). 2016**. *Plano de Ação Nacional para a Conservação dos Mamíferos da Mata Atlântica Central*. ICMBio. Brasília, Brazil.

**Estrada A, Raboy BE, Oliveira LC.** **2012**. Agroecosystems and primate conservation in the tropics: A review. *American Journal of Primatology* **74**:696–711.

**Ferreira LV, Lemos de Sá R, Buschbacher, Batmanian G, Cardoso da Silva JM, Arruda MB, Moretti E, S.N. de Sá LF, Falcomer J, Bampi MJ.** **2001**. Identificação de áreas prioritárias para a conservação da biodiversidade por meio da representatividade das unidades de conservação e tipos de vegeteção nas ecorregiões da Amazônia brasileira. Pages 268–286 in A. Veríssimo, A. Moreira, D. Sawyer, I. dos Santos, L. P. Pinto and J. P. R Capobianco, editors. *Biodiversidade na Amazonia Brasileira*, Editora Estação Liberdade, Instituto Socioambiental, São Paulo, Brasil.

**Fialho MS, Ludwig G, Valença-Montenegro MM.** **2016**. Legal international trade in live Neotropical Primates originating from South America. *Primate Conservation* **30**:1-6.

**Fobissie K. 2015.** Landscape approaches in the Congo Basin: linking the Democratic Republic of Congo’s Emission Reduction Program (ERP) and the Central Africa Regional Program for the Environment (CARPE). In: Minang PA, van Noordwijk, M, Freeman OE, Mbow C, de Leeuw J, Catacutan D. eds. *Climate-Smart Landscapes: Multifunctionality in Practice*. Nairobi, Kenya: World Agroforestry Centre (ICRAF), 361-371. Available at www.asb.cgiar.org/climate-smart-landscapes/chapters/chapter25.pdf

**Ganzhorn JU.** **1987**. A possible role of plantations for primate conservation in Madagascar. *American Journal of Primatology* **12**:205–215.

**Ganzhorn, JU, Abraham JP.** **1991**. A possible role of plantations for lemur conservation in Madagascar: food for folivorous species. *Folia* *Primatologica* **56**:171–176.

**Gardner CJ, Davies ZG.** **2014**. Rural bushmeat consumption within multiple-use protected areas: qualitative evidence from southwest Madagascar. *Human Ecology* **42***:*21–34.

**GFW, 2018.** Global Forest Watch**.** Available at http://www.globalforestwatch.org/country/COD.

**Gilardi K, Gillespie T, Leendertz F, Macfie E, Travis D, Whittier C, Williamson E.** 2015. Best Practice Guidelines for Health Monitoring and Disease Control in Great Ape Populations (IUCN/SSC Primate Specialist Group). Available at www.primate-sg.org/ best_practice_disease.

**Global Invasive Species Database. 2018.** Species profile: *Macaca fascicularis*. Available at http://www.iucngisd.org/gisd/speciesname/Macaca%20fascicularis on 03-02-2018.

**Gogarten JF, Calvignac-Spencer S, Leendertz FH. 2017**. Ebola virus disease. In: Fuentes A, ed. *The International Encyclopedia of Primatology*. John Wiley and Sons. DOI: 10.1002/9781119179313.wbprim0390.

**Golden CD. 2009**. Bushmeat hunting and use in the Makira Forest, north-eastern Madagascar: a conservation and livelihoods issue. *Oryx* **43**:386-392.

**Golden CD, Gupta AC, Vaitla B, and Myers SS. 2016**. Ecosystem services and food security: assessing inequality at community, household and individual scales. *Environmental Conservation* **43**:381-388. 10.1017/s0376892916000163

**Harich FK, Treydte AC. 2016**. Mammalian wildlife diversity in rubber and oil palm plantations CAB Reviews 2016 0, No. 020 Available at http://www.cabi.org/cabreviews.

**Hending D, Andrianiaina A, Rakotomalala Z, Cotton S.** **2017**. Range extension and behavioural observations of the recently described Sheth's dwarf lemur (*Cheirogaleus shethi*). *Folia Primatolica* **88**:401-408

**Hicks TC, Darby L, Hart J, Swinkels J, January N, Menken S. 2010**. Trade in orphans and bushmeat threatens one of the Democratic Republic of the Congo's most important populations of eastern chimpanzees (*Pan troglodytes schweinfurthii*). *African Primates* **7**:1-18.

**Hilser H, Sampson H, Melfi V, Tasirin JS. 2013**. Sulawesi crested black macaque Macaca nigra Species Action Plan: Draft 1. *Selamatkan Yaki–Pacific Institute, Manado, Indonesia*. Available at www.psgb.org/pdfs/Regine_Gross_final_report_small.pdf.

**Hockings KJ, McLennan, MR, Carvalho S, Ancrenaz M, Bobe R, Byrne RW, Dunbar RIM, Matsuzawa T, McGrew WC, Williamson EA, Wilson ML, Wood B, Wrangham RW, Hill CM. 2015**. Apes in the Anthropocene: flexibility and survival. *Trends in Ecology & Evolution* **30**:215–222.

**Holst B, Medici E, Marinho-Filho O, Kleiman D, Leus K, Pissinatti A, Vivekananda G, Ballou J, Traylor-Holzer K, Raboy B, Passos FC, Vleeschouwer K, Valença-Montenegro MM. 2006**. Lion tamarin population and habitat viability assessment workshop 2005: final report. *IUCN/SSC Conservation Breeding Specialist Group*. Apple Valley, USA. 208. Available at [www.icmbio.gov.br/...de.../PAN_Mamíferos_da_Mata_Atlântica_Central_RED1_Parte2](http://www.icmbio.gov.br/...de.../PAN_Mamíferos_da_Mata_Atlântica_Central_RED1_Parte2)

**INEA. 2015**. *Centro de Primatologia do Rio de Janeiro*. Instituto Estadual do Ambiente. Rio de Janeiro, Brasil. 292.

**ITTO, 2009.** *DRC cancels nearly 60% of timber contracts*, In Tropical Timber Market Report: ITTO Market Information Service. p. 2. International Tropical Timber Organization (ITTO), Yokohama, Japan. Available at <http://www.itto.int/>

**IUCN.** **2017**. International Union for Conservation of Nature, IUCN Red List of

Threatened Species. Version 2017-3–4; [www.iucnredlist.org](http://www.iucnredlist.org).

**Jenkins RKB, Keane A, Rakotoarivelo AR, Rakotomboavonjy V, Randrianandrianina FH, Razafimanahaka HJ, Ralaiarimalala SR, Jones JPG.** **2011**. Analysis of patterns of bushmeat consumption reveals extensive exploitation of protected species in Eastern Madagascar. *PLOS ONE* **6**: e27570.

**Jerusalinsky L, Talebi M, Melo FR (orgs.).** **2011**. *Plano de Ação Nacional para a Conservação dos Muriquis* – *Brachyteles arachnoides* e *Brachyteles hypoxanthus*. ICMBio. Brasília, Brasil. 141.

**Jerusalinsky L. 2016.** Emerging challenges for Brazilian primate conservation: perspectives from the strategic planning. *Scientific* *Program of the XXVI Congress of the International Primatological Society*. Abstract #6515

**Jones-Engel L, Engel GA, Schillaci MA, Rompis A, Putra A, Suaryana KG, Fuentes A, Beer B, Hicks S, White R, Wilson B. 2005.** Primate-to-human retroviral transmission in Asia. *Emerging Infectious Diseases***11:**1028-1035**.**

**Kierulff MCM, Santos GR, Canale GR, Carvalho C, Cassano C, Gouveia P, Gatto C. 2005.** *Plano de Manejo para a conservação do macaco-prego-do-peito-amarelo Cebus xanthosternos*. Unpublished report. Instituto de Estudos Socioambientais do Sul da Bahia. Ilhéus, Brasil*.* 45.

**Kierulff MCM. 2010.** Invasive introduced golden-headed lion tamarins - a new threat to golden lion tamarins. *Tamarin Tales* **10**:5-7.

**Kierulff MCM, Ruiz-Miranda CR, de Oliveira PP, Beck BB, Martins A, Dietz JM, Rambaldi DM, Baker AJ. 2012**., The Golden lion tamarin *Leontopithecus rosalia*: a conservation success story. *International Zoo Yearbook* **46**:36–45.

**KSBK.** **2002**. Dibalik perdagangan dagin primata di Lampung Sumatera. KSBK, Malang. Available at http://www.profauna.net/id/tentang-profauna/apa-itu-profauna#.WmwhH6iWbIU**Lee RJ, Gorog AJ, Dwiyahreni A, Siwu S, Riley J, Alexander H, Paoli GD, Ramono W. 2005**. Wildlife trade and implications for law enforcement in Indonesia: a case study from North Sulawesi. *Biological Conservation* **123**:477-488.

**Maldonado AM, Nijman V, Bearder SK.** **2009**. Trade in night monkeys *Aotus* spp. in the Brazil–Colombia–Peru tri-border area: international wildlife trade regulations are ineffectively enforced. *Endangered Species Research* **9**:143-149.

**McLennan MR, Spagnoletti N, Hockings KJ. 2017**. The implications of primate behavioral flexibility for sustainable human–primate coexistence in anthropogenic habitats. *International Journal of Primatology* **38**:105–121

**Meijaard E, Wich S, Ancrenaz M, Marshall A J.** **2012**. Not by science alone: Why orangutan conservationists must think outside the box. *Annals of the New York Academy of Sciences* **1249**:29–44.

**Melo FR, Ferraz DS, Valença-Montenegro MM, Oliveira LC, Pereira DG, Port-Carvalho M. 2015.** Avaliação do risco de extinção de *Callithrix aurita* (É. Geoffroy, 1812) no Brasil. Processo de avaliação do risco de extinção da fauna brasileira. ICMBio. Brazil. Website: http:// [www.icmbio.gov.br/portal/biodiversidade/fauna-](http://www.icmbio.gov.br/portal/biodiversidade/fauna-) brasileira/estado-de conservacao/7198-mamiferos-callithrix-aurita-sagui-da-serra-escuro. html.

**Merker S, Yustian I, Mühlenberg M.** **2005**. Responding to forest degradation: altered habitat use by Dian’s tarsier *Tarsius dianae* in Sulawesi, Indonesia. *Oryx* **39**: 189–195.

**Michon G, de Foresta H.** **1995**. The Indonesian agro-forest model. In: Halladay P, Gimour DA, eds. *Conserving Biodiversity Outside Protected Areas: The Role of Traditional Agroecosystems*. IUCN, Gland. 90–106.

**Mittermeier RA, Robles Gil P, Mittermeier CG.** **1997**. Megadiversity: Earth’s Biologically Wealthiest Nations. (CEMEX, Mexico City, Mexico).

**Mittermeier, RA, Fonseca GAB da, Rylands AB, Brandon K. 2005**. A brief history of biodiversity conservation in Brazil. *Conservation Biology* **19**:601–607.

**Mockrin MH, Bennett EL, Labruna DT. 2005**. Wildlife farming: a viable alternative to hunting in tropical forests? Wildlife Conservation Society, Bronx, NY. Available at <https://library.wcs.org/DesktopModules/Bring2mind/DMX/Download.aspx>?

**Molinario G, Hansen MC, Potapov PV, Tyukavina A, Stehman S, Barker B, Humber M. 2017.** Quantification of land cover and land use within the rural complex of the Democratic Republic of Congo. *Environmental Research Letters* **12**:104001.

**Molinario G, Hansen MC, Potapov PV. 2015**. Forest cover dynamics of shifting

cultivation in the Democratic Republic of Congo: a remote sensing-based assessment for 2000–2010. *Environmental Research Letters* **10**:094009 DOI:10.1088/1748-9326/10/9/094009.

**Morgan D, Mundry R, Sanz C, Ayina CE, Strindberg S, Lonsdorf E, Kühl HS. 2017.** African apes coexisting with logging: Comparing chimpanzee (*Pan troglodytes troglodytes*) and gorilla (*Gorilla gorilla gorilla*) resource needs and responses to forestry activities. *Biological Conservation*. <https://doi.org/10.1016/j.biocon.2017.10.026>.

Mukul SA, Saha N. 2017. Conservation benefits of tropical multifunctional land-uses in and around a forest protected area of Bangladesh. *Land* **6** (1) doi:10.3390/land6010002

**Musibono DE, Kabangu F, Munzundu A, Kisangala M, Nsimanda I, Sinikuna M, Kileba A.** **2010**. Les différents traités environnementaux sont-ils appropriés pour les populations des pays en développement (Afrique)? *VertigO* - *la revue électronique en sciences de l’environnemen*t. Available at http://vertigo.revues.org/9398.

**Nantha HS, Tisdell C.** **2009**. The orangutan–oil palm conflict: economic constraints and opportunities for conservation. *Biodiversity and Conservation* **18**:487–502.

**Nasi R, Taber A, Van Vliet N.** **2011**. Empty forests, empty stomachs? Bushmeat and livelihoods in the Congo and Amazon Basins. *International Forestry Review* **13**:355-368.

**Nater, A., Mattle-Greminger MP, Nurcahyo A, Nowak MG., de Manuel M, Desai T, Groves C, Pybus M, Sonay Tugce B, Roos C, Lameira AR., Wich SA., Askew J, Davila-Ross M, Fredriksson G, de Valles G, Casals F, Prado-Martinez J, Goossens B, Verschoor EJ, Warren KS., Singleton I, Marques DA., Pamungkas J, Perwitasari-Farajallah D, Rianti P, Tuuga A, Gut IG., Gut M, Orozco-terWengel P, van Schaik CP, Bertranpetit J, Anisimova M, Scally A, Marques-Bonet T, Meijaard E, and Krützen M.** **2017**. Morphometric, behavioral, and genomic evidence for a new orangutan species. *Current Biology* **27**:1–12.

**Nekaris KAI.** **2016**. The Little Fireface Project: Community conservation of Asia’s slow lorises via ecology, education, and empowerment. In: Waller M, ed. *Ethnoprimatology*. *Developments in Primatology: Progress and Prospects* Springer International Publishing, 259-272.

**Nekaris KAI, McCabe S, Spaan D, Ali MI, Nijman V.** **2018**. A novel application of cultural consensus models to evaluate conservation education programs. *Conservation Biology 32:466-476* DOI:10.1111/cobi.13023.

**Nekaris KAI, Shepherd CR, Starr CR, Nijman V.** **2010**. Exploring cultural drivers for wildlife trade via an ethnoprimatological approach: a case study of slender and slow lorises (*Loris* and *Nycticebus*) in South and Southeast Asia. *American Journal of Primatology* **72**:877-886.

**Newmark WD, Jenkins CN, Pimm SL, McNeally PB, Halley JM.** **2017**. Targeted habitat restoration can reduce extinction rates in fragmented forests. *Proceedings of the National Academy of Siences* **114**:9635–9640 [DOI:10](http://www.pnas.org/lookup/suppl/doi:10).1073/pnas.1705834114.

**Nijman V.** **2009**. *An assessment of trade in gibbons and orang-utans in Sumatra, Indonesia*. Kuala Lumpur. TRAFFIC Southeast Asia.

[www.trafficj.org/publication/09_Assessment_Trade_Gibbons_Orang-utans.pdf](http://www.trafficj.org/publication/09_Assessment_Trade_Gibbons_Orang-utans.pdf)

**Nijman V, Martinez CFY, Shepherd CR.** **2009**. Saved from trade: donated and confiscated gibbons in zoos and rescue centres in Indonesia. *Endangered Species Research* **9**:151-157.

**Nijman V, Spaan D, Rode‐Margono EJ, Nekaris KAI.** **2017**. Changes in the primate trade in Indonesian wildlife markets over a 25‐year period: Fewer apes and langurs, more macaques, and slow lorises. *American Journal of Primatology* **79**:e22517 DOI: 10.1002/ajp.22517.

**Nlandu Mayamba T. 2012.** *Mapping Police Services in the Democratic Republic of Congo: Institutional Interactions at Central, Provincial and Local Levels.* Institute of Development Studies Research Reports: Institute of Development Studies. p 103. Avaialable at https://www.ids.ac.uk/files/dmfile/rr71.pdf

**Nogueira DM, Ferreira AMR, Goldschmidt B, Pissinatti A, Carelli JB, Verona CE. 2011.** Cytogenetic study in natural hybrids of *Callithrix* (Callitrichidae: Primates) in the Atlantic forest of the state of Rio de Janeiro, Brazi. Iheringia: *Série Zoologia* **101**:156–160.

**Norris D, Rocha-Mendes F, Marques R, Almeida Nobre R, Galetti M. 2011.** Density and spatial distribution of buffy-tufted-ear marmosets (*Callithrix aurita*) in a continuous Atlantic forest. *International Journal of Primatology* **32**:811–829.

**Nunn CL, Gillespie TR. 2016.** Infectious disease and primate conservation. In: Wich SA, Marshall AJ, eds. *An introduction to primate conservation*. Oxford University Press, 157-173.

**Oliveira PP, Grativol AD, Ruiz-Miranda CR. 2008.** *Conservação do mico-leão-dourado: enfrentando os desafios de uma paisagem fragmentada*. Associação Mico-Leão-Dourado & Editora da UENF. Campos dos Goytacazes, Brazil. 1-199.

**Oliveira LC, Grelle CEV.** **2012**. Introduced primate species of an Atlantic Forest region in Brazil: present and future implications for the native fauna. *Tropical Conservation Science* **5**:112-120.

**Pereira DG, Oliveira MEA, Ruiz-Miranda CR. 2008**. Interações entre calitriquídeos exóticos e nativos no Parque Nacional daSerra dos Órgãos-RJ. *Revista Espaço E Geografia* **11**:87–114.

**Plumptre, A., Nixon, S., Caillaud, D., Hall, J.S., Hart, J.A., Nishuli, R. & Williamson, E.A. 2016**. *Gorilla beringei ssp. graueri*. The IUCN Red List of Threatened Species.

**Port-Carvalho M, Kierulff MCM. 2009.** *Callithrix aurita* (É. geoffroy, 1812) primates, callitrichidae. In: Bressan PM, Kierulff MCM, Sugieda AM, eds. *Fauna Ameaçada de Extinção no Estado de São Paulo: Vertebrados (Vol. 1)*. São Paulo: Fundação Parque Zoológico de São Paulo e Secretaria do Meio Ambiente.

**Quinten MF, Stirling S, Schwarze Y, Dinata K, Hodges K. 2014**. Knowledge, attitudes and practices of local people on Siberut Island (West-Sumatra, Indonesia) towards primate hunting and conservation. *Journal of Threatened Taxa* **6**:6389-6398; DOI: 10.11609/jot.o3963.6389-98.

**Pedersen AB, Altizer S, Poss M, Cunningham AA, Nunn CL. 2005**. Patterns of host specificity and transmission among parasites of wild primates. *International Journal for Parasitology* **35**:647-657.

**Prance GT. 1990. Consensus for conservation. *Nature* 345: 384.**

**Raboy B, Neves L, Zeigler S, Saraiva N, Cardoso N, Santo G, Ballou J, Leimgruber P. 2010**. Strength of habitat and landscape metrics in predicting golden-headed lion tamarin presence or absence in forest patches. *Biotropica* **42**:388–397.

**Raharivololona BM, Ganzhorn JU. 2009. Gastrointestinal parasite infection of the gray mouse lemur (Microcebus murinus) in the littoral forest of Mandena, Madagascar: effects of forest fragmentation and degradation. *Madagascar Conservation and Development* 4: 103-112**

**Rambaldi DM, Kleiman DG, Mallinson JJC, Dietz LA, Padua SM. 2008.** O papel das Organizações Não-Governamentais e do Comitê Internacional para a Conservação e Manejo de *Leontopithecus* na conservação do mico-leão. In: Kleiman DG, Rylands AB, eds. *Micos-leões: biologia e conservação.* *Ministério do Meio Ambiente. Brasília, Brasil,* 105-135.

**Rambaldi DM, Baker AJ**. **2012**. The golden lion tamarin *Leontopithecus rosalia*: A conservation success story. *International Zoo Yearbook*. **46**:36–45.

**Razafimanahaka JH, Jenkins RK, Andriafidison D, Randrianandrianina F, Rakotomboavonjy V, Keane A, Jones JP.** **2012**. Novel approach for quantifying illegal bushmeat consumption reveals high consumption of protected species in Madagascar. *Oryx*, **46**:584-592.

**Reuter KE, Gilles H, Wills AR, Sewall BJ. 2016**. Live capture and ownership of lemurs in Madagascar: extent and conservation implications. *Oryx*, **50:**344-354**Reuter KE,** **Schaefer MS. 2016**. Captive conditions of pet lemurs in Madagascar. *Folia Primatologica* **87**:48-63.

**Rezende GC. 2014**. *Mico-leão-preto: a história de sucesso na conservação de uma espécie ameaçada.* Matrix. São Paulo, Brasil. 1-176

**Ribeiro MC, Metzger JP, Martensen AC, Ponzoni FJ, Hirota MM.** **2009**. The Brazilian Atlantic Forest: How much is left, and how is the remaining forest distributed? Implications for conservation. *Biological Conservation* **142**:1141–1153.

**Rittera CD., McCratec G, Nilssona RH, Fearnsided PM, Palmef U, Antonellia A. 2017**. Environmental impact assessment in Brazilian Amazonia: Challenges and prospects to assess biodiversity. *Biological Conservation* **206**:161–168.

**Robinson JG, Bennett EL. 2000**. *Hunting for sustainability in tropical forests*. Columbia University Press, New York. p. 582.

**Ruiz-Miranda CR, Affonso, Morais MM, Verona CES, Martins A, Beck BB. 2006.** Behavioral and ecological interactions between reintroduced golden lion tamarins (*Leontopithecus rosalia* Linnaeus, 1766) and introduced marmosets (*Callithrix* spp, Linnaeus, 1758) in Brazil’s Atlantic Coast Forest fragments. *Arquivos de Biologia e Tecnologia* **49**:99-109.

**Rylands AB.** **1990**. Priority areas for conservation in Amazonia. *Trends in Ecology and Evolution* **5**:240-241.

**Rylands AB., Brandon K.** **2005**. Brazilian protected areas. *Conservation Biology* **19**:612–618.

**Rylands AB. Huber O. Brown Jr. KS.** **1991**. *Workshop-90, Biological Priorities for Conservation in Amazonia*. Map scale 1:5,000,000. Instituto Brasileiro do Meio-Ambiente e dos Recursos Naturais Renováveis (Ibama), Brasília, Instituto Nacional de Pesquisas da Amazônia (INPA), Manaus, and Conservation International, Washington, DC.

**Rylands AB, Strier KB, Mittermeier RA, Borovansky J, Seal US.** **1998**. *Population and Habitat Viability Assessment (PHVA) for the Muriqui (Brachyteles arachnoides).* IUCN/SSC Conservation Breeding Specialist Group (CBSG). Apple Valley, USA. 1-122.

**Salafsky N.** **1993**. Mammalian use of a buffer zone agroforestry system bordering Ganung Palung National Park, West Kalimantan, Indonesia. *Conservation Biology* **7**:928–933.

**Schwitzer N, Clough D, Zahner H, Kaumanns W, Kappeler P, Schwitzer C. 2010.** Parasite prevalence in blue-eyed black lemurs *Eulemur flavifrons* in differently degraded forest fragments. *Endangered Species Research* **12**:215-225.

**Semroc B, Thomas M, Ward J, Buchanan J. 2015.** *Incentivizing No-Deforestation Palm Oil Production in Liberia and the Democratic Republic of Congo*. Washington, D.C., USA: USAID-supported Forest Carbon, Markets and Communities Program. p 58.

**Setiawan A, Nugroho TS, Wibisono Y, Ikawati V, sugardjito J. 2012.** Population density and distribution of Javan gibbon (*Hylobates moloch*) in Central Java, Indonesia. *Biodiversitas Journal of Biological Diversity*. **1:**23-27.

**Setiawan A, Wibisono Y, Nugroho TS, Agustin IY, Imron MA, Pudyatmoko S. Javan Surili**. **2010**. A survey population and distribution in Mt. Slamet Central Java, Indonesia. *Journal Primatology Indonesia* **7:**51-54.

**Shekelle, M, Salim A.** **2009**. An acute conservation threat to two tarsier species in the Sangihe Island chain, North Sulawesi, Indonesia. *Oryx*, **43:**419-426.

**Shepherd CR.** **2010**. Illegal primate trade in Indonesia exemplified by surveys carried out over a decade in North Sumatra. *Endangered Species Research* **11**:201-205.

**Simmen B, Bayart F, Marez A, Hladik A.** **2007**. Diet, nutritional ecology, and birth season of *Eulemur macaco* in an anthropogenic forest in Madagascar. *International Journal of Primatology* **28**:1253–1266.

**Stiles D.** **1998**. The Mikea hunter-gatherers of southwest Madagascar: Ecology and socioeconomics. *African Studies Monographs* **19**:127-148.

**Strier KB.** **2007**. *Faces na Floresta*. Sociedade para a Preservação do Muriqui – Preserve Muriqui. Rio de Janeiro, Brail. 1-190.

**Struebig MJ, Fischer M, Gaveau DL, Meijaard E, Wich SA, Gonner C, Sykes R, Wilting A, Kramer-Schadt S.** **2015**. Anticipated climate and land-cover changes reveal refuge areas for Borneo’s orang-utans. *Global Change Biology* **21**:2891–2904.

**Svensson MS, Ingram DJ, Nekaris KAI, Nijman V.** **2015**. Trade and ethnozoological use of African lorisiforms in the last 20 years. *Hystrix, the Italian Journal of Mammalogy* **26**:153-161.

**Talebi MG, Melo FR, Dias LG, Cunha AA, Mendes SL, Breves P, Jerusalinsky L.** **2011**. Contextualização sobre *Brachyteles arachnoides* e *Brachyteles hypoxanthus.* In: Jerusalinsky L, Talebi M, Melo FR, eds. *Plano de Ação Nacional para a conservação dos muriquis*. Instituto Chico de Conservação da Biodiversidade – ICMBio, Série Espécies Ameaçadas n^o^ 11. Brasília, Brasil. 16-61.

**Trefon T. 2009.** Public Service Provision in a Failed State: Looking Beyond Predation in the Democratic Republic of Congo. *Review of African Political Economy* **36**:9-21.

**Trefon T. 2010.** Administrative obstacles to reform in the Democratic Republic of Congo. *International Review of Administrative Sciences* **76**:702-722.

**Trefon T. 2013.** Uncertainty and powerlessness in Congo 2012. *Review of African Political Economy* **40**:141-151.

**Trefon T. 2016**. *Congo's Environmental Paradox; Potential and Predation in a Land of Plenty*. USA: Zed Books.

**Trevelin LC, Port-Carvalho M, Silveira M, Morell E. 2007**. Abundance, habitat use and diet of *Callicebus nigrifrons Spix* (primates, Pitheciidae) in Cantareira State Park, S˜ao Paulo, Brazil. *Revista Brasileira do Zoologia* **24**:1071–1077.

**USAID. 2012.** US Agency for International Development (US AID)/Central Africa Regional Program for the Environment (CARPE) Regional Development Cooperation Strategy 2012–2020, 1–38.

**USDA Brazil Soya.** **2017**. World Agricultural Production. United States Department of Agriculture Foreign Agricultural Service Circular Series WAP 05-17 May 2017. Available at https://apps.fas.usda.gov/psdonline/circulars/production.pdf.

**Van Vliet N, Mesa MPQ, Cruz-Antia D, de Aquino LJN, Moreno J, Nasi R.** **2014**. The uncovered volumes of bushmeat commercialized in the Amazonian trifrontier between Colombia, Peru and Brazil. *Ethnobiology and Conservation* **3**:1-11.

**Van Vliet N, Nebesse, C, Gambalemoke S, Akaibe D, Nasi R. 2012**. The bushmeat market in Kisangani, Democratic Republic of Congo: implications for conservation and food security. *Oryx*, **46:**196-203.

**Vasconcelos PFC. 2017**. Yellow fever. In: Marcondes, CB, ed*. Arthropod borne diseases*. Springer, 101-113.

**Warren-Thomas E, Dolman PM, Edwards DP.** **2015**. Increasing demand for natural rubber necessitates a robust sustainability initiative to mitigate impacts on tropical biodiversity. *Conservation Letters* **8**:230–24.

**Wetterberg GB., Jorge Pádua, MT, Castro CS de, Vasconcellos., JMC de.** **1976**. Uma análise de prioridades em conservação da natureza na Amazônia. Projeto de Desenvolvimento e Pesquisa Florestal (PRODEPEF), PNUD/FAO/IBDF/BRA-45, Série Técnica 8:63pp**.**

**Wetterberg GB, Prance GT, Lovejoy TE.** **1981**. Conservation progress in Amazonia: a structural review. *Parks* **6**:5-10.

**Wilkie DS, Curran B, Tshombe R, Morelli GA. 1998**. Managing bushmeat hunting in Okapi Wildlife Reserve, Democratic Republic of Congo. *Oryx* **32**:131-144.

**Wilkie DS, Wieland M., 2015.** Conserving and Eating Wildlife in Africa. WCS Working Papers **47**, 50.

**Wilkie DS, Wieland M, Boulet H, Le Bel S, van Vliet N, Cornelis D, BriacWarnon V, Nasi R, Fa JE. 2016**. Eating and conserving bushmeat in Africa. *African Journal of Ecology* **54**:402-414.

**World Bank. 2017.** http://data.worldbank.org/country (accessed 11 January 2018).

**Yanggen D, Angu K, Tchamou N. 2010.** *Landscape-Scale Conservation in the Congo Basin : Lessons Learned from the Central Africa Regional Program for the Environment (CARPE).* Gland, Switzerland: IUCN.

**Ziegler S, Fa JE, Wohlfart C, Streit B, Jacob S, Wegmann M. 2016.** Mapping bushmeat hunting pressure in Central Africa. *Biotropica* **48:**405–412.

**Zimkus BM, Lawson LP, Barej MF, Barratt CD, Channing A, Dash KM, Dehling JM, Du Preez L, Gehring P-S, Greenbaum E, Gvoždík V, Harvey J, Kielgast J, Kusamba C, Nagy ZT, Pabijan M, Penner J, Rödel M-O, Vences M, Lötters S.** **2017**. Leapfrogging into new territory: How Mascarene ridged frogs diversified across Africa and Madagascar to maintain their ecological niche. *Molecular Phylogenetics and Evolution* **106**:254-269.
